# Antimicrobial, antiproliferative activities and molecular docking of metabolites from *Alternaria alternata*

**DOI:** 10.1186/s13568-023-01568-1

**Published:** 2023-07-06

**Authors:** Heba T. Khazaal, Mohamed T. Khazaal, Ahmed S. Abdel-Razek, Ahmed A. Hamed, Hassan Y. Ebrahim, Reham R. Ibrahim, Mokhtar Bishr, Yara E. Mansour, Rabab A. El Dib, Hesham S. M. Soliman

**Affiliations:** 1 Department of Pharmacognosy, Faculty of Pharmacy, Helwan University, Ain-Helwan, Cairo, 11795 Egypt; 2 Botany and Microbiology Department, Faculty of Science, Helwan University, Cairo, 11795 Egypt; 3 Microbial Chemistry Department, National Research Center, 33 El-Buhouth Street, Giza, 12622 Egypt; 4 Plant General Manager and Technical Director of the Arab Company for Pharmaceuticals and Medicinal, Plants, Cairo, Egypt; 5 Pharmaceutical Organic Chemistry Department, Faculty of Pharmacy, Helwan University, Ain-Helwan, Cairo, 11795 Egypt; 6 PharmD program, Egypt-Japan University of Science and Technology (E-JUST), New Borg El-Arab City, Alexandria, 21934 Egypt

**Keywords:** *Colocasia esculanta* leaves, *Alternaria alternata*, Antimicrobial activity, Human prostatic adenocarcinoma, Molecular docking

## Abstract

Endophytic fungi allied to plants have sparked substantial promise in discovering new bioactive compounds. In this study, propagation of the endophytic fungus *Alternaria alternata* HE11 obtained from *Colocasia esculanta* leaves led to the isolation of Ergosterol (1), *β*-Sitosterol (2), Ergosterol peroxide (3), in addition to three dimeric naphtho-γ-pyrones, namely Fonsecinone A (4), Asperpyrone C (5), and Asperpyrone B (6), which were isolated from genus *Alternaria* for the first time. Structures of the isolated compounds were established on the basis of extensive 1D and 2D NMR and, MS measurements. The ethyl acetate extract, as well as compounds 1, 3, 4 and 6 were evaluated for their antimicrobial activity using agar well-diffusion and broth microdilution assays. Molecular docking study was carried out to explore the pharmacophoric moieties that governed the binding orientation of antibacterial active compounds to multidrug efflux transporter AcrB and the ATP binding site to *E. coli* DNA gyrase using MOE software. Results revealed that the most active antibacterial compounds 4 and 6 bind with high affinity in the phenylalanine-rich cage and are surrounded with other hydrophobic residues. The antiproliferative activity of all isolated compounds was in vitro evaluated using the human prostatic adenocarcinoma cell lines DU-145, PC-3, PC-3 M, 22Rv1 and CWR-R1ca adopting MTT assay. Compound 4 was the most active against almost all tested cell lines, with IC_50_ values 28.6, 21.6, 17.1 and 13.3 against PC-3, PC-3 M, 22Rv1 and CWR-R1ca cell lines, respectively.

**Graphical Abstract:**

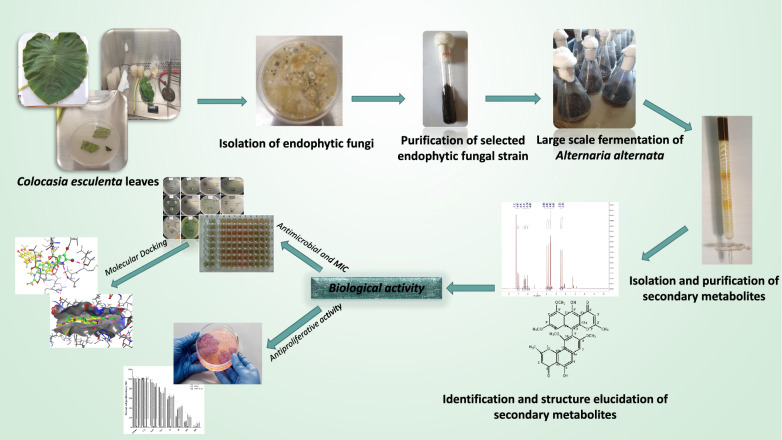

**Supplementary Information:**

The online version contains supplementary material available at 10.1186/s13568-023-01568-1.

## Key points

- Characterization of Alternaria alternata HE11 strain from Colocasia esculenta leaves
- First isolation of three dimeric naphtho-γ-pyrones from genus Alternaria
- Fonsecinone A and Asperpyrone B showed significant anti-prostate cancer and antibacterial activity, respectively

**Supplementary Information:**

The online version contains supplementary material available at 10.1186/s13568-023-01568-1.

## Introduction

Currently, the world is facing severe health problems that present life-threat to millions of people. Antimicrobial resistance and cancer are among these life-threatening health issues. Antimicrobial resistance (AMR) presents a worldwide health concern and a development threat rendering about 700,000 deaths yearly. Drug resistance could provoke 10 million deaths annually by 2050. If this problem continues without action, the success of modern medicine in combating infections arising from major surgeries and cancer chemotherapy would be at increased risk (WHO, 2019 and 2021).

Cancer is a leading cause of death worldwide, accounting approximately 10 million deaths in 2020, for nearly one in six deaths; prostate cancer is considered one of the most common cancer types with about 1.41 million cases in 2020 (WHO, 2022).

Endophytes are plant-associated microorganisms that populate the internal tissues of plant species without causing disease symptoms or apparent changes and became a promising source of novel bioactive compounds. (Samanta et al. 2021). In contrast to the low productivity and vulnerability of plants as sources of novel metabolites, endophytes are considered a quickly renewable, reproducible source with a limitless supply of bioactive metabolites, particularly endophytic fungi, which have been recognized as a significant source of active metabolites with potential antimicrobial, anti-inflammatory, antioxidant, immunosuppressive, as well as cytotoxic activities (Chandra 2012; Uzma et al. 2018; Vasundhara et al. 2019). Genus *Alternaria* is a member of family Pleosporaceae and comprises about 378 species (Li et al. 2022) reported to produce diverse classes of bioactive secondary metabolites, including pyrones, terpenoids, nitrogen-containing compounds, steroids, phenolics, and quinones with variable biological activities, including antimicrobial, antioxidant, antiviral, anticancer, and phytotoxic activities (Abd Elghaffar et al. 2022; Tsuge et al. 2013; Bräse et al. 2009; Lou et al. 2013).

As part of our ongoing research on chemical constituents with important therapeutic properties of endophytic fungi isolated from plants, we herein report the isolation and full taxonomical characterization of *Alternaria alternata* HE11 strain from the leaves of *Colocasia esculenta* L. for the first time. The fungal isolate was subjected to large-scale fermentation, followed by different chromatographic and spectroscopic procedures, resulting in the isolation and identification of six secondary metabolites from the ethyl acetate soluble fraction (EtOAc) of the fungal isolate. Furthermore, the EtOAc extract and isolated compounds were evaluated for their antimicrobial and antiproliferative activity against five prostatic cell cancer.

## Materials and methods

### Isolation and culture of endophytic fungi

The fungal strain *Alternaria alternata* HE11 was isolated from *Colocasia esculanta* L. leaves, which were collected from Al Monier Village, Al-Sharkya Governorate, Egypt. Based on its morphological features, the plant was identified by Prof. Emad Farahat, Professor of Plant Ecology, Faculty of Science, Helwan University, labeled and transported in an icebox to the National Research Center laboratory. The identified specimens were launched in the Microbial Chemistry Lab., Biotechnology Research Institute, National Research Center, Cairo, Egypt, and deposited at the Pharmacognosy Department, Faculty of Pharmacy, Helwan University (33*Ces*1/2018). In accordance with Kyeremeh et al. (2017) method, endophytic fungi were isolated from plant parts. To make sure that the isolated fungi were endophytes, the surfaces of plant parts were sterilized through rinsing with sterile distilled water (SDW), then immersed in 70% ethanol for 1 min. After that the pieces were yet again rinsed using SDW, and dipped in 2% sodium hypochlorite for 1 min, followed by rinsing with SDW triply. The sterilized plant parts were dried in laminar flow, and a healthy leaf was sliced into small pieces of around 1 cm^2^ and placed on a potato dextrose agar plate (PDA; potato extract: dextrose: bacteriological agar; 4.0:20.0:15.0 g/L; CONDA, Madrid. Spain) at pH 5.6 ± 0.2. The efficiency of the surface sterilization procedure was checked through inoculation of 4–5 water drops from the final rinse on a PDA medium and held for about 5–6 days to investigate the growth of any endophytic fungi. The identification of the most potent obtained fungal endophyte was performed using 18S rRNA analysis, Macrogen Company, South Korea. The identified fungal isolate, *Alternaria alternata* HE11 strain, was deposited at the Egypt Microbial Culture Collection (EMCC, CAIRO MIRCEN), Ain Shams University, which is a part of the World Data Centre for Microorganisms (WDCM) (https://ccinfo.wdcm.org/details?regnum=583) with the accession code, EMCC 99965 (accessed on 01 March 2023).

### Identification of the isolated endophytic fungi

#### Phenotypic identification

The fungal isolate was preliminarily identified using cultural and morphological features such as colony growth pattern and conidial morphology (Woudenberg et al. 2013).

#### Genotypic identification

For further confirmation of identification, 18S rDNA sequence analysis was performed for the fungal isolate. For fungal DNA extraction, mycelia were inoculated in a 250 mL Erlenmeyer’s flask containing 50 mL of potato dextrose broth medium and incubated for 4 days at 28 °C. After the incubation period, the mycelial biomass was harvested and the genomic DNA was isolated using the Qiagen DNeasy Mini Kit, USA following the manufacturer’s manual (Abdelgawad et al. 2022). The amplification reactions of the 18S rRNA gene were performed using 2 universal primers, namely NS3 (5′-GCAAGTCTGGTGCCAGCAGCC amplification 3′)/NS4 (5′-CTTCCGTCAATTCCTTTAAG-3′) (Lu et al. 1995). The following PCR thermal profile was used: denaturation step at 94 °C for 5 min, then 35 cycles of 94 °C for 30 s, 55 °C for 30 s, 72 °C for 90 s, and finally an extension step at 72 °C for 5 min. The amplified products were examined by electrophoresis and sequenced in SolGent Company, South Korea. The sequence produced was analyzed using BLAST online tool to study the similarity and homology of the 18S rRNA gene sequences with the similar existing sequences available at NCBI database (National Centre Biotechnology Information, http://www.ncbi.nlm.nih.gov). MEGAX software was used for construction of the phylogenetic tree.

#### Large-scale fermentation of endophytic fungi

Suspensions of the selected isolated fungi were inoculated into Erlenmeyer flasks containing 100 mL of yeast malt extract medium (ISP2; malt extract: yeast extract: glucose; 10.0:4.0:4.0 g/L; Sigma-Aldrich), the pH was adjusted at 7.2, and cultivated as seed culture for 3 days at 28 °C. Five millimeters of the seed culture were used to inoculate 250 mL Erlenmeyer flasks containing modified rice medium composed of 100 g commercial rice and 150 mL of distilled water containing 0.4% yeast extract and 1% malt extract, under sterile conditions. The flasks were incubated for two weeks at 37 °C; after harvesting, the obtained cultures were macerated in methanol, followed by filtration under a vacuum. For complete removal of the methanol, the resulting aqueous methanol filtrate was then evaporated using a rotary evaporator at 40 °C. The remaining water residue was re-extracted by vigorous shaking with ethyl acetate. Finally, the obtained ethyl acetate extract was concentrated *in vacuo* to dryness yielding the desired organic extract (11 g) (Mahmoud et al. 2018).

#### Isolation and purification of secondary metabolites

Isolation and purification of secondary metabolites contained in the EtOAc extract were performed using different chromatographic techniques. A part of the crude extract (10 g) was chromatographed over silica gel G 60 column (70–230 mesh, E. Merck, Germany; 3 × 60 cm) eluting successively with cyclohexane:DCM:MeOH (*v/v*, 100:0:0, 50:50:0, 0:100:0, 0:98:2, 0:95:5, respectively) to afford five collectively fractions (I–V) based on their TLC profile (precoated Silica gel 60 F_254_ aluminum sheets; 0.2 mm thickness; E. Merck, Darmstadt, Germany) after visualization under UV light (254 and 365 nm) and spraying with *p*-Anisaldehyde/ H_2_SO_4_ reagent (Oleszek et al. 2008), followed by heating to give different colours. Fraction I (650 mg) eluted by 100% cyclohexane, revealed constituents of limited interest. Fraction III (eluted with 50% cyclohexane/DCM, 2 g) was re-chromatographed over a silica gel column (100 g) eluting with cyclohexane:EtOAc gradients (*v/v* 100:0–80:20), to afford two collective subfractions (A1 and A2; 30, 25 mg, respectively). Subfraction A1 and A2 were individually applied on Sephadex LH-20 columns (25–100 μm, Pharmacia, Uppsala, Sweden) eluting with 60% DCM/MeOH to afford compound 1 (12 mg) and compound 2 (15 mg), respectively. Fraction IV (eluted with 98% DCM/MeOH, 2.5 g) was re-chromatographed over silica gel column (125 g) eluted with DCM:EtOAc gradients (*v/v* 100:0—80:20), to give 2 collective subfractions B1 (50 mg) and B2 (1 g). Subfraction B1 was purified using Sephadex LH-20 column eluted with 60% DCM/MeOH to afford compound 3 (30 mg), while subfraction B2 was applied on silica gel column using DCM:EtOAc gradients (*v/v* 100:0–80:20), to yield pure compound 4 (25 mg), in addition to collective subfraction C1 (90 mg). The latter was separated using PTLC (Silica gel 60 F_254_ glass plates, 0.5 mm thickness, Merck, Darmstadt, Germany) and 85% DCM/EtOAc as mobile phase, to afford compound 5 (14 mg) and compound 6 (18 mg), respectively.

#### Spectroscopic and spectrometric analyses of isolated compounds

1D (^1^H and ^13^C) NMR and 2D (HMQC and HMBC) NMR spectra were recorded on a JEOL Eclipses ECS-400 NMR spectrometer (JEOL in., Peabody, MA, USA) at 400 MHz for ^1^H NMR and 100 MHz for ^13^C NMR, using the residual solvent peak ( H = 7.26 and  C = 77.1, CDCl_3_) as a reference. ESI–MS positive ion acquisition mode was carried out on a XEVO TQD triple quadruple instrument; Waters Corporation, Milford, MA01757 U.S.A, mass spectrometer; using ACQUITY UPLC—BEH C18 1.7 µm–2.1 × 50 mm Column with flow rate: 0.2 mL\min, and solvent system consisted of (A) Water containing 0.1% formic acid and (B) Acetonitrile.

### In vitro antimicrobial activity study

#### Antimicrobial susceptibility test

The study was carried out for the crude EtOAc extract, as well as for the isolated compounds 1, 3, 4 and 6 using agar well-diffusion assay, according to the Clinical and Laboratory Standards Institute (CLSI 2019) and Gholizadeh et al. (2013). The test microbes were obtained from American Type Culture Collection (ATCC, USA) and included the four Gram’s positive bacteria *Listeria monocytogenes* (ATCC 7644), *Clostridium perfringens* (ATCC 13124), *Staphylococcus aureus* (ATCC 25923) and *Streptococcus faecalis* (ATCC 8043); the four Gram’s negative bacteria *Escherichia coli* (ATCC 8739)*, Pseudomonas aeruginosa* (ATCC 9027)*, Klebsiella pneumoniae* (ATCC 700603) and *Salmonella enterica* (ATCC 14028), in addition to the Fungi *Candida albicans* (ATCC 10231) and the *Aspergillus niger* (ATCC 6275). Conventional antibiotics with different modes of action (Bioanalyse, Turkey), including Amikacin (AK 30) as a protein synthesis inhibitor; Amoxicillin (AX 25) and Ampicillin/sulbactam (SAM 20), as cell wall synthesis inhibitors; Norfloxacin (NOR 10) and Ofloxacin (OFX 5), as DNA replication inhibitors and Nystatin (NS 50 µg/mL) as an antifungal for disruption of the cell membrane, were used as reference drugs, while 10% DMSO was used as a negative control. For the study 100 µl of the microbial suspension (Optical density, OD = 0.2) containing 1 × 10^5^ cells of each reference strain were seeded individually with Muller Hinton (MH) agar plates, after solidification of the poured medium, 0.6 cm diameter wells were made. Each well received 50 µl of a tested sample, then the plates were kept in the refrigerator for the diffusion of the tested samples (Shinde et al. 2021). The plates were then incubated at 37 °C for 24 h and at 25 °C for 5 days for inoculated bacterial and fungal strains, respectively. The antimicrobial susceptibility of each extract was determined by measuring the diameter of the developed inhibition zones in mm.

#### Determination of minimum inhibitory concentrations (MIC)

The assay was carried out for the EtOAc extract, as well as for the isolated compounds 1, 3, 4 and 6 using broth microdilution assay, according to the European Committee on Antimicrobial Susceptibility Testing (European Committee on Antimicrobial Susceptibility Testing (EUCAST) of the European Society of Clinical Microbiology and Infectious Diseases (ESCMID) 2003), Balouiri et al. (2016) and CLSI (2019). Stock solutions were prepared by dissolving 32 mg of the EtOAc extract, 4 mg of compound 1, and 3 mg of each of the compounds 3,4 and 6 in 1 mL DMSO. One hundred microliters of sterile MHB were added in each well (2–12), while 150 µl of each stock sample were added in the first column of microtiter plates. A two-fold dilution for each stock sample was prepared by transferring 100 µl from the first to the 11th well. One hundred microliters of each of the ten tested microbial inoculums containing 1 × 10^5^ CFU/mL (OD = 0.08–0.12 at 625 nm) were transferred to each well, except for the last one which was used as blank. All microtiter plates were incubated at 37 °C for 24 h and 25 °C for 5 days for inoculated bacterial and fungal strains, respectively; Elisa reader was used for recording the results. The antifungal compounds nystatin (NS; 0.2 mg/ml) and fluconazole (FCZ; 0.2 mg/ml) along with antibacterial chloramphenicol (1 mg/ml) and ciprofloxacin (1 mg/ml) were used as positive controls.

#### Molecular docking study

The compounds tested in the antimicrobial study were built using MOE 2014.0901 and filed in a molecular database file (ULC 2018). The crystal structure of the Bacterial AcrB efflux pump and DNA gyrase B with the inhibitor (MBX and 6G9) were downloaded from the protein data bank (PDB) ID: 5ENO (Sjuts et al. 2016) and 5L3J (Gjorgjieva et al. 2016), respectively. Protein was energy minimized and 3D protonated via the structure preparation module of MOE. The complexed ligand and water molecules were removed from the crystal structure before conducting docking. The site of docking was recognized and the database containing all the tested compounds has been docked using rigid receptor as a docking protocol and triangle matcher as a placement method. London dG and GBVI/WSA dG were selected as rescoring functions and the force field was used as a refinement. Free binding energy (kcal/mol) was calculated and only the best-scored pose was obtained for each compound. The docked pose with the highest docking score has been recognized as the most probable binding conformation of the ligand within the binding site.

#### In vitro cell proliferation assay

The human prostatic adenocarcinoma cell lines DU-145, PC-3, PC-3 M, 22Rv1 were purchased from the American Type Culture Collection (ATCC, Manassas, VA, USA). The castration-resistant CWR-R1ca cells were provided by Dr. Elmageed, Edward Through College of Osteopathic Medicine, Monroe, Louisiana, USA. All cancerous cells were maintained in RPMI-1640 (Corning, Manassas, VA, USA), supplemented with 10% fetal bovine serum (R&D Systems, Inc., Minneapolis, MN, USA), 2 mM L-glutamine and 1% antibiotic/antimycotic solution (Corning, Manassas, VA, USA). Cells were cultured at 37 °C and 5% CO_2_ in a humidified incubator (VWR, Radnor, PA, USA), and were checked periodically for cell growth and confluency. At 80–90% confluent monolayers, cells were washed with phosphate buffered saline (PBS) and detached by 0.05% trypsin/EDTA (Corning, Manassas, VA, USA) for 5–10 min, and cell pellets were collected by centrifugation for subsequent cell culturing (Riss et al. 2016); Docetaxel was used as reference drug.

### Statistical analysis

One-way ANOVA and simple linear regression were used to analyze the data obtained from the antimicrobial study by using GraphPad Prism Version 9.0 for Windows (GraphPad Software Inc., San Diego, CA). In vitro anticancer cell proliferation screening assays were performed at least two times in triplicates as per indicated compound concentrations. The statistical analysis was executed by one-way ANOVA followed by Tukey’s post hoc test for multiple comparisons between mean cell proliferation at indicated concentrations and DMSO-treated control cells.

## Results

### Phenotypic identification of the endophytic fungus

**Fig. 1:**
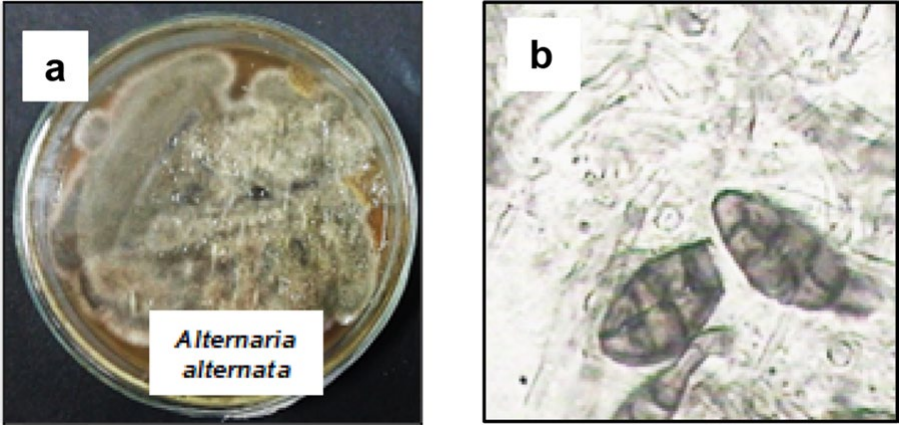
Endophytic fungus *Alternaria alternata* HE11. **a** Colony on potato dextrose agar plate (PDA); **b** Light microscopical examination of mycelium (400X)

The dark grey colonies (Fig. 1a) of the fungus *Alternaria alternata* HE11 were fast growing, attaining a diameter of 5.0 cm on malt extract agar in 7 days. The mycelium was septate and the conidia were variable in shape from ovoid to obclavate varying from 25 ~ 38 to 7.5 ~ 16.5 µm in size. The conidiophores aroused directly from the hyphae, developed abundantly and were 5.0 μm in size. The morphological results of the isolated fungus confirmed the isolated pathogen as *Alternaria sp.* Light microscopical examination of mycelium is presented in Fig. 1b.

### Genotypic identification of the endophytic fungus

**Fig. 2:**
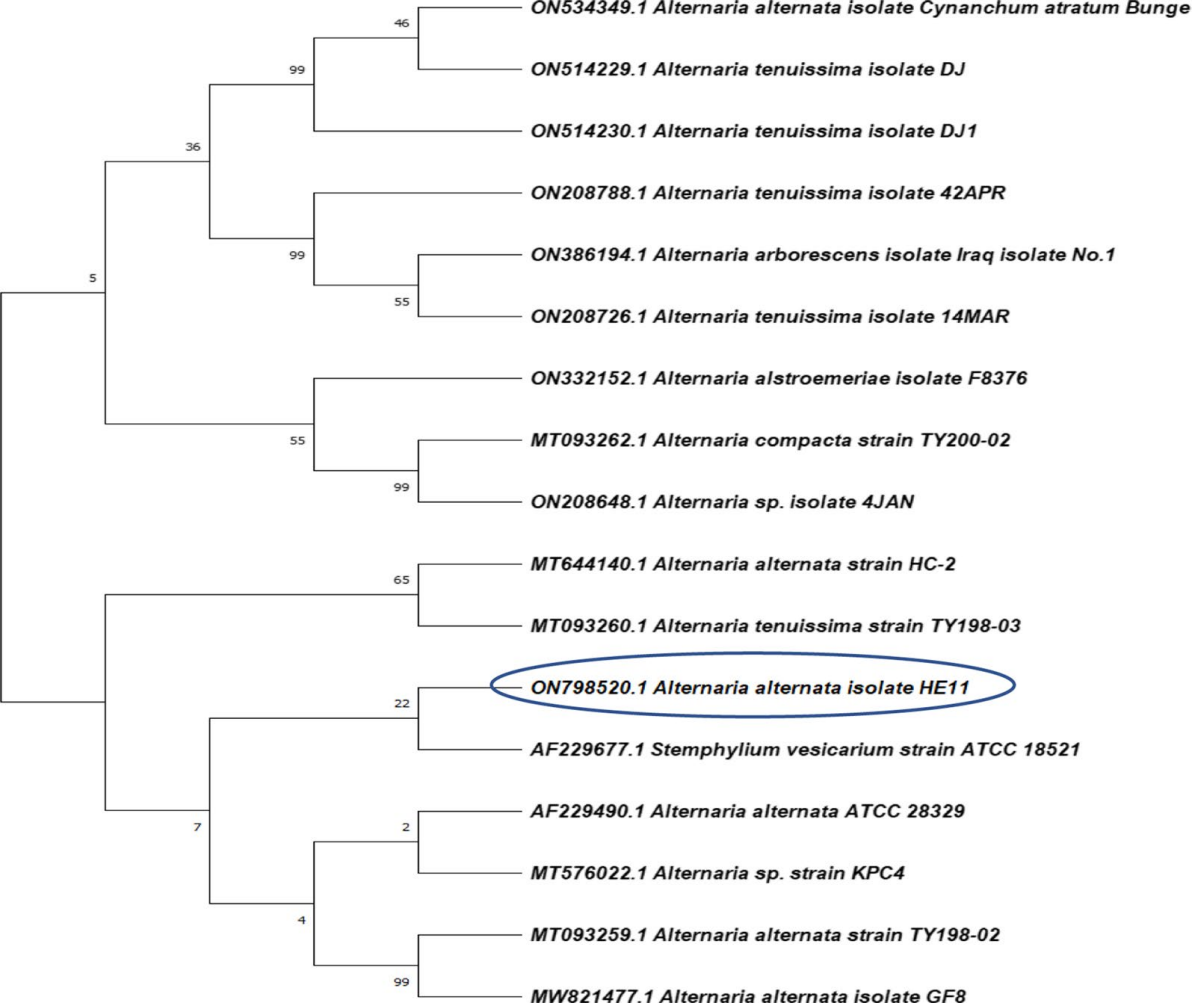
Neighbor-joining phylogenetic tree of HE11 strain based on 18S rRNA gene sequences, showing its close relationship to *Alternaria alternata* species

The 18S rRNA gene sequence was utilized to genetically identify the isolated stain and oppose other identified sequences existing in the database of GenBank using BLAST to specify the similarities score in addition to calculate the statistically significant differences of matches (http://www.blast.ncbi.nlm.nih.gov/Blast). The results established a very close similarity with *Alternaria alternata* using the 18S rRNA gene sequence with 100% homology of the isolate HE11. The Neighbor-Joining approach was used to infer the evolutionary history (Saitou and Nei 1987). In the bootstrap test (500 repetitions), the proportion of duplicate trees in which the linked taxa clustered together is displayed next to the branches (Felsenstein 1985). With branch lengths in the same units as the evolutionary distances used to infer the phylogenetic tree, the tree is drawn to scale. The evolutionary distances calculation was performed using the Maximum composite Likelihood method (Tamura et al. 2004) and are in the units of the number of base substitutions per site. Fifteen nucleotide sequences were used in the investigation and codon positions were 1st, 2nd, 3rd and Noncoding. For each sequence pair, all unclear places were eliminated (pairwise deletion option). The resulting dataset contained 552 locations in total, and MEGA X was used to conduct evolutionary analysis (Kumar et al. 2018). Based on the analysis of the DNA sequence and morphological characteristics of the HE11 isolate, the isolated strain was identified as *Alternaria alternata* strain HE11 and deposited in GenBank with the accession no. ON798520.1. The phylogenetic tree of *Alternaria alternata* HE11 strain is shown in Fig. 2.

### Characterization of isolated compounds

Compounds 1 and 2 were obtained as white crystals. *R*_f_ = 0.44 and 0.46; respectively (*v/v*, 95:5, DCM/ MeOH), Examination of the NMR data of compounds 1 and 2, and by comparison with the reported literature, they were identified as Ergosterol (Martinez et al. 2015) and *β*-Sitosterol (Yoo et al. 2006); respectively (Additional file 1: Fig. S1–S3 for compound 1 and S4–S5 for compound 2).

Compound 3 was isolated as white crystals. *R*_f_ = 0.65 (*v/v*, 7:3, cyclohexane/EtOAc), neither-UV absorbing nor fluorescent, giving a deep violet or blue colour upon spraying with *p*-anisaldehyde/sulfuric acid. It has the molecular formula C_28_H_44_O_3_ ([M-H]^+^ at *m/z* 429.3561). ^1^H NMR (400 MHz, CDCl_3_, δ in ppm, *J* in Hz): δ 6.49 (1H, d, *J* = 8.8 Hz, H-7), 6.23 (1H, d, *J* = 8.4 Hz, H-6), 5.20 (1H, dd, *J* = 7.6, 15.2 Hz, H-23), 5.11 (1H, dd, *J* = 7.6, 15.2 Hz, H-22), 3.95 (1H, m, H-3), 0.98 (3H, d, *J* = 6.8 Hz, CH_3_-21), 0.89 (3H, d, *J* = 6.8, CH_3_-28), 0.86 (3H, s, CH_3_-19), 0.81 (3H, d, *J* = 6.8 Hz, CH_3_-27), 0.79 (3H, s, CH_3_-18), 0.78 (3H, d, *J* = 6.4 Hz, CH_3_-26). ^13^C NMR (100 MHz, CDCl_3_): δ 135.5 (C-6), 135.3 (C-22), 132.4 (C-23), 130.8 (C-7), 82.3 (C-5), 79.5 (C-8), 66.5 (C-3), 56.2 (C-17), 51.7 (C-9), 51.1 (C-14), 44.6 (C-13), 42.8 (C-24), 39.9 (C-20), 39.4 (C-12), 37.0 (C-4), 37.0 (C-10), 34.8 (C-1), 33.1 (C-25), 30.2 (C-2), 28.8 (C-16), 23.5 (C-15), 21.0 (C-11), 20.7 (C-27), 20.1 (C-26), 19.7 (C-21), 18.3 (C-19), 17.7 (C-28), 13.0 (C-18) (Additional file 1: Fig. S6–S10).

**Fig. 3:**
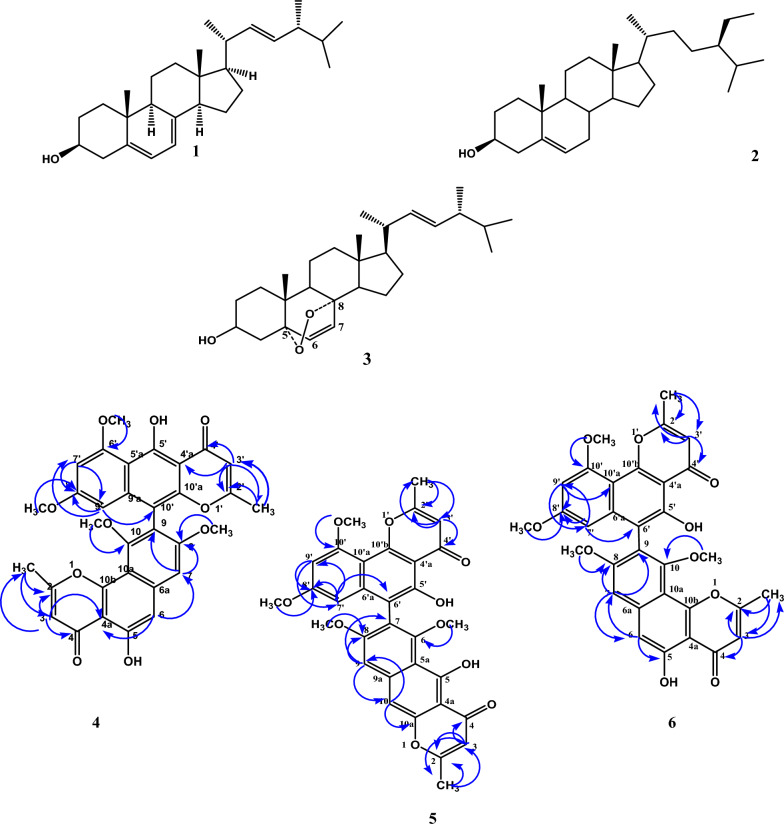
Chemical structures of compounds isolated from the endophytic fungus *Alternaria alternata*. Ergosterol (1), *β*-Sitosterol (2), Ergosterol peroxide (3), Fonsecinone A (4), Asperpyrone C (5), and Asperpyrone B (6), showing observed key HMBC correlations of compounds 4, 5 and 6

Compound 4 was isolated as a yellow powder, *R*_f_  = 0.42 (*v/v*, 9:1, DCM/EtOAc), showing a dark spot under UV light (245/365 nm) and giving an orange colour upon spraying with *p*-anisaldehyde/sulfuric acid spray reagent. Compound 5 was isolated as a yellow powder, *R*_f_  = 0.58 (*v/v*, 9:1, DCM/EtOAc), showing a dark spot under UV light (245/365 nm) and giving yellow colour upon spraying with *p*-anisaldehyde/sulfuric acid reagent. Compound 6_,_ was isolated as a yellow powder, *R*_f_  = 0.54 (*v/v*, 9:1, DCM/EtOAc), showing a dark spot under UV light (245/365 nm) and giving yellow colour upon spraying with *p*-anisaldehyde/sulfuric acid spray reagent. Structures of the isolated compounds are presented in Fig. 3; ^1^H and^13^C-NMR, and HMBC spectral data, and correlations of compounds 4, 5, and 6 (CDCl_3_, δ in ppm, *J* in Hz) are compiled in Table 1 and shown in Fig. 3 and Additional file 1: Fig. S11–S24.

**Table 1:** ^1^H and^13^C-NMR, and HMBC spectral data and HMBC correlations of compounds 4, 5 and 6 (CDCl_3_, δ in ppm, *J* in Hz)

Compound 4				Compound 5			Compound 6		
Position	δ_H_ (mult., *J* in Hz)	Δc	HMBC	δ_H_ (mult., *J* in Hz)	δc	HMBC	δ_H_ (mult., *J* in Hz)	δc	HMBC
2		167.6			167.7			167.0	
3	6.33 (1H, s)	110.8	2-CH_3_, 2, 4 and 4a	6.02 (1H, s)	107.5	2-CH_3_, 2, 4 and 4a	6.31 (1H, s)	110.7	2-CH_3_, 2, 4 and 4a
4		183.1			184.6			183.0	
4a		109.5			104.7			109.4	
5		156.8			160.9			156.5	
5a					111.6				
6	7.04 (1H, s)	106.1	4a, 5, 7 and 10´b		158.3		7.02 (1H, s)	106.3	4a, 5 and 7
6a		140.9						140.8	
7	6.96 (1H, s)	101.7	8, 9, 10 and 10a		118.4		6.97 (1H, s)	102.0	6, 8 and 9
8		160.1			160.3			160.0	
9		117.2		6.97 (1H, s)	101.8	5a,7, 8 and 10		118.0	
9a					140.7				
10		157.0		7.11 (1H, s)	101.4	4a, 5a, 9 and 10a		156.7	
10a		108.1			153.3			108.2	
10b		155.2						155.1	
2-CH_3_	2.47 (3H, s)	20.7	2 and 3	2.39 (3H, s)	20.9	2 and 3	2.47 (3H, s)	20.7	2 and 3
6-OCH_3_				3.62 (3H, s)	62.3	6			
8-OCH_3_	3.77 (3H, s)	56.1	8	3.80 (3H, s)	56.2	8	3.79 (3H, s)	56.2	8
10-OCH_3_	3.41 (3H, s)	61.4	10				3.60 (3H, s)	55.3	10
2´		167.0			166.6			166.7	
3´	6.00 (1H, s)	107.5	2`-CH_3_, 2`, 4` and 4`a	6.31 (1H, s)	110.4	2´-CH_3_, 2´, 4´ and 4´a	6.32 (1H, s)	110.4	2`-CH_3_, 2`, 4` and 4`a
4´		184.7			183.1			183.1	
4´a		104.4			108.4			108.5	
5´		162.9			154.3			154.4	
5`a		108.7							
6´		161.2			110.0			109.7	
6´a					140.8			140.9	
7´	6.41 (1H, d, J = 2.4)	97.1	5`a, 8` and 9`	6.24 (1H, d, *J* = 2)	96.7	6´, 8´, 9´ and 10´a	6.18 (1H, d, *J* = 2.4)	96.4	6`, 8`, 9` and 10`a
8´		161.7			161.5			161.6	
9´	6.17 (1H, d, J = 2.4)	96.3	5`a, 7`, 8` and 10`	6.41 (1H, d, *J* = 2)	96.8	7´, 8´ and 10´a	6.43 (1H, d, *J* = 2.4)	96.8	7`, 8` and 10`a
9`a		140.7							
10´		105.1			159.5			159.6	
10´a		150.9			105.1			105.1	
10´b					155.5			155.9	
2´-CH_3_	2.11 (3H, s)	20.9	2` and 3`	2.53 (3H, s)	20.7	2´ and 3´	2.54 (3H, s)	20.7	2` and 3`
6´-OCH_3_	4.02 (3H, s)	56.4	6`						
8´-OCH_3_	3.60 (3H, s)	55.3	8’	3.60 (3H, s)	55.3	8´	3.60 (3H, s)	61.6	8`
10´-OCH_3_				3.98 (3H, s)	56.1	10´	3.99 (3H, s)	56.1	10`

### Antimicrobial activity study

The antimicrobial activity of the EtOAc extract and four isolated compounds (1, 3, 4, and 6) from *Alternaria alternata* HE11 were determined qualitatively through agar well-diffusion assay by measuring zone of inhibition (ZOI) diameters in mm (Table 2 and Additional file 1: Fig. S25–S29) and quantitatively by estimating MICs (Table 3).

**Table 2:** Antimicrobial susceptibility test results of the EtOAc extract, and compounds 1, 3, 4 and 6 from the endophytic *Alternaria alternata strain* HE11

Samples and Reference Drugs	EtOAC extract			1			3			4			6			AK 30	AX 25	SAM 20	NOR 10	OFX 5	NS
	1	0.75	0.5	1	0.75	0.5	1	0.75	0.5	1	0.75	0.5	1	0.75	0.5						
Gram-positive bacteria																					
* C. perfringens* ATCC 13124	14	12	10	13	12	10	16	14	11	14	13	11	15	13	10	23	18	11	34	30	NT
* L. monocytogenes* ATCC 7644	–	–	–	–	–	–	–	–	–	–	–	–	–	–	–	7	7	7	8	7	NT
* S. aureus* ATCC 25923	–	–	–	10	9	9	12	10	7	–	–	–	13	11	10	22	21	24	25	27	NT
* S. faecalis* ATCC 8043	9	9	8	9	9	9	11	10	8	12	11	11	12	1	11	22	21	24	25	27	NT
Gram-negative bacteria																					
* E. coli* ATCC 8739	12	11	9	12	11	9	9	8	8	11	11	10	11	10	10	22	–	19	30	29	NT
* K. pneumoniae* ATCC 700603	13	12	9	13	12	10	–	–	–	12	10	9	13	10	9	21	16	10	30	30	NT
* P. aeruginosa* ATCC 9027	14	13	10	14	13	12	13	11	10	14	13	11	15	14	11	21	–	–	30	20	NT
* S. enterica* ATCC 14028	14	13	11	14	12	11	–	–	–	11	10	8	–	–	–	21	21	10	35	30	NT
Fungi																					
* A. niger* ATCC 6275	17	13	9	17	14	10	–	–	–	–	–	–	–	–	–	NT	NT	NT	NT	NT	–
* C. albicans* ATCC 10231	–	–	–	–	–	–	14	12	9	–	–	–	–	–	–	NT	NT	NT	NT	NT	–

Results represent the mean of the clear zone of inhibition for three replicates diameter (mm) of each trial; All concentrations are expressed in mg/mL; (–), Not detected; (NT), Not tested; *Amikacin* (AK 30), protein synthesis inhibitor; *Amoxicillin* (AX 25) and *Ampicillin/sulbactam* (SAM 20), cell wall synthesis inhibitors, *Norfloxacin* (NOR 10) and *Ofloxacin* (OFX 5), DNA replication inhibitor; *Nystatin* (NS 50 µg/mL), antifungal for disruption of the cell membrane; Diluted DMSO was used as a negative control

**Table 3:** Minimum inhibitory concentrations (MIC) for EtOAc extract and the isolated compounds 1, 3, 4 and 6

Samples reference strains	EtOAc extract*	1**	3***	4*	6*	C	CIP	NS	FCZ
Gram-positive bacteria									
* C. perfringens* ATCC 13124	11.5	> 4	3	3	1.5	0.5	0.5	NT	NT
* L. monocytogenes* ATCC 7644	> 23	> 4	> 3	> 3	> 3	0.5	0.25	NT	NT
* S. aureus* ATCC 25923	> 23	> 4	> 3	> 3	> 3	0.25	0.5	NT	NT
* S. faecalis* ATCC 8043	> 23	> 4	> 3	> 3	> 3	0.25	0.125	NT	NT
Gram-negative bacteria									
* E. coli* ATCC 8739	11.5	> 4	> 3	> 3	> 3	0.5	0.25	NT	NT
* K. pneumoniae* ATCC 700603	11.5	> 4	> 3	> 3	> 3	0.25	0.25	NT	NT
* P. aeruginosa* ATCC 9027	5.75	> 4	> 3	> 3	0.75	0.25	0.5	NT	NT
* S. enterica* ATCC 14028	5.75	> 4	> 3	> 3	> 3	0.25	0.25	NT	NT
Fungi									
* A. niger* ATCC 6275	0.719	2	> 3	> 3	> 3	NT	NT	0.05	0.025
* C. albicans* ATCC 10231	> 23	> 4	> 3	> 3	> 3	NT	NT	0.025	0.0125

All values are in mg/mL

*P-*Value * = 0.0000000; ** = 0.0000011; *** = 0.0000002; (NT), Not tested; antibacterial positive controls Chloramphenicol (C, 1 mg/ml); Ciprofloxacin (CIP, 1 mg/ml); antifungal positive controls Nystatin (NS; 0.2 mg/ml); Fluconazole (FCZ, 0.2 mg/ml)

Among the tested strains, *Clostridium perfringens* and *Pseudomonas aeruginosa* were the most susceptible to all tested samples, while *Listeria monocytogenes* was the most resistant tested bacteria, followed by the fungus *Candida albicans*.

The EtOAc extract significantly inhibited the growth of all tested Gram’s negative bacteria, with MIC values 5.75 mg/mL against *Pseudomonas aeruginosa* and *Salmonella enterica*, and 11.5 mg/mL against *Escherichia coli* and *Klebsiella pneumoniae*. The extract inhibited only the growth of the Gram’s positive bacteria *Clostridium perfringens* with MIC value 11.5 mg/mL, while the MIC value for the fungus *Aspergillus niger* was 0.719 mg/mL. As compared to the EtOAc extract, compound 6 was more effective against *Pseudomonas aeruginosa* and *Clostridium perfringens,* with MICs values 0.75 and 1.5 mg/mL, respectively. In addition, compounds 3 and 4 were more effective against *Clostridium perfringens* than the EtOAc extract, with MICs value 3 mg/mL. Finally, compound 1 was the only active antifungal compound against *Aspergillus niger* (MIC = 2 mg/mL). Results of the antimicrobial susceptibility test and Minimum Inhibitory Concentration (MIC) study for the EtOAc extract, and compounds 1, 3, 4, and 6 are compiled in Tables 2 and 3, respectively.

### Identification of the possible antibacterial targets of isolated compounds by molecular docking

**Fig. 4:**
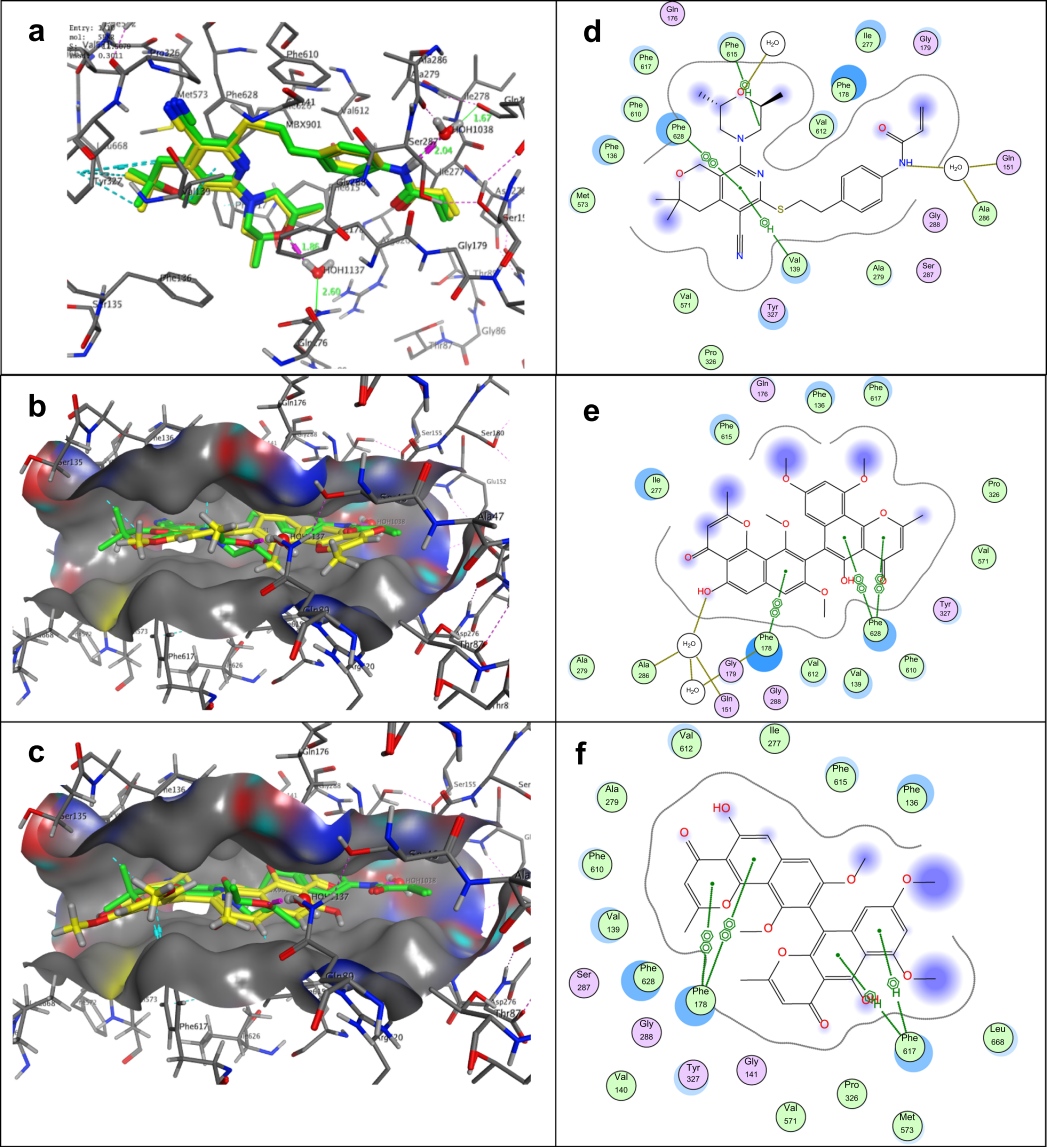
**a** Validation process: superimposition of the co-crystallized ligand (green) and the redocked cocrystallized ligand MBX3135 (yellow) into the AcrB binding sites (PDB ID: 5ENR); **b** 3D-structure of Compound 6 and MBX3135; **c** 3D-structure of Compound 4 and MBX3135. Co-crystallized ligand MBX3135 with green colour and the docked molecule with yellow colour; **d**, **e** and **f** 2D-structure interaction poses of AcrB-MBX3135, compounds 6 and 4, respectively

**Fig. 5:**
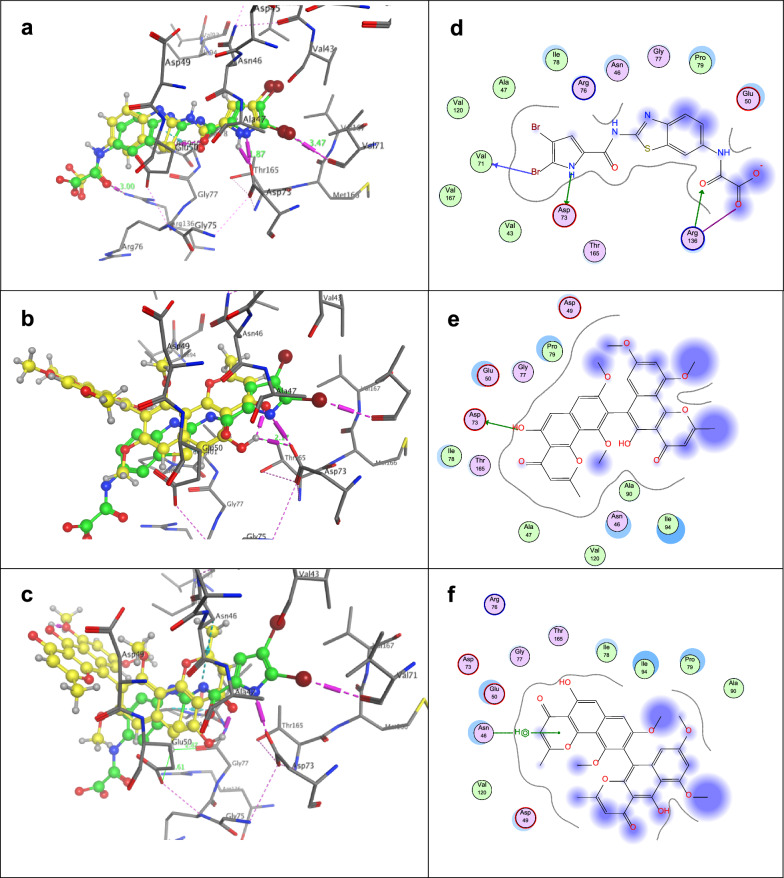
**a** Validation process: superimposition of the co-crystallized ligand (green) and the re-docked co-crystallized ligand 6G9 (yellow) into the ATP binding site of DNA gyrase B enzyme (PDB ID: 5L3J); **b** 3D-structure of Compound **6** and 6G9; **c** 3D-structure of Compound 4 and 6G9. Co-crystallized ligand 6G9 with green colour and the docked molecule with yellow colour; **d**, **e** and **f** 2D-structure interaction poses of GyrB-6G9, compounds 6 and 4, respectively

Molecular docking studies were carried out to understand the mechanistic aspects of the antibacterial analogues 1, 3, 4 and 6, to predict the binding orientation with multidrug efflux transporter AcrB and the ATP binding site to *E. coli* DNA gyrase B, and to explore the detailed intermolecular interactions for determining the probable binding mode; this was conducted using Molecular Operating Environment software (MOE, 2014.0901). Results of the molecular docking study are compiled in Table 4 and presented in Figs. 4 and 5.

**Table 4:** Results of molecular docking of the Crystallized Ligand and most active compounds versus (PDBID: 5ENO) and PDB ID: 5L3J

Ligand/Compound	PDB ID: 5ENO			PDB ID: 5L3J		
	Score (kcal/mol)	RMSD (Å)	Interacting amino acids	Score (kcal/mol)	RMSD (Å)	Interacting amino acids
Crystallized Ligand	− 11.50	0.30	Val139∼3.4 Å Phe615∼4 Å Phe628∼3.5 Å H_2_O (OH) = 2.04 Å Gln151(H_2_O) = 1.67 Å	− 6.01	0.95	Arg136 = 3.0 Å Asp73 = 1.87 Å Val 71 = 3.47 Å
1	− 7.68	1.40	Phe615∼3.3 Å	− 5.36	1.21	–
3	− 5.99	0.84	Phe615∼3.2 Å	− 5.10	1.70	Arg136 = 2.17 Å
4	− 8.84	0.46	Phe617∼3.3 Å Phe178∼3 Å	− 5.93	1.52	Glu50 = 2.61 Å Gly77 = 2.87 Å
6	− 9.46	0.51	Phe628∼4.3 Å Phe178∼2.8 Å H_2_O (OH) = 1.86 Å Gln151(H_2_O) = 1.67 Å	− 6.09	0.71	Asp73 = 2.17 Å

### Cell proliferation assay

To assess the effect of isolated metabolites on the proliferation of different human prostate cancer cell lines, the MTT-based cell proliferation assay was adopted, where DU-145, PC-3, PC-3 M, 22Rv1, and CWR-R1ca cells were incubated with different concentrations of tested metabolites for 48 h.

As shown in Table 5, isolated endophytic metabolites exhibited concentration–response inhibition against all tested prostate cancer cell lines, with calculated IC_50_ values ranging from low three digits to low two digits µM. All tested compounds exhibited significant antiproliferative activity (P < 0.001) against all tested prostate cancer cell lines starting from concentration 12.5 µM for compounds 1, 4, and 5, and from 25 µM for compounds 3 and 6, and finally 100 µM for compound 2. Compound 4 showed the highest antiproliferative activity of all tested compounds against the three cell lines CWR-R1ca, 22Rv1 cells and PC-3, with IC_50_ values 13.3, 17.,1 and 28.6 µM, respectively, as well as IC_50_ value 21.6 µM against PC-3 M cell line. Furthermore, compound 1 was the most active against PC-3 M and DU-145 cells, with IC_50_ 19.3 and 32.9 µM, respectively, in addition to 21.6 µM against CWR-R1ca. Finally, compounds 5, 3 and 6 showed lower activity against all test cell lines, with IC_50_ ranges of 22.8–46.7, 35.2–49.5 and 61.4–136.7 µM, respectively; while compound 2 was the least active with IC_50_ range from 101.6 to above 200 µM. The results of the cell proliferation assay are compiled in Table 5 and shown in Additional file 1: Fig. S30–S31).

**Table 5:** Calculated IC_50_ values (Mean ± DS) of the isolated metabolites 1–6 against different prostate cancer cell lines in the proliferation assay

Compound	DU-145	PC-3	PC-3 M	22Rv1	CWR-R1ca
1	32.9 ± 3.5	44.7 ± 5.3	19.3 ± 3.0	32.2 ± 4.5	21.6 ± 4.4
2	> 200	> 200	136.6 ± 5.5	101.6 ± 6.5	122.7 ± 7.3
3	35.2 ± 4.2	47.6 ± 3.6	39.7 ± 4.0	41.0 ± 2.8	49.5 ± 4.6
4	41.1 ± 6.5	28.6 ± 6.2	21.6 ± 1.7	17.1 ± 3.9	13.3 ± 2.3
5	46.7 ± 6.3	31.7 ± 6.6	22.8 ± 3.7	28.9 ± 5.2	38.4 ± 5.6
6	136.7 ± 6.9	70.2 ± 3.9	92.2 ± 5.0	61.4 ± 6.0	78.1 ± 4.9

All values are listed in μM

## Discussion

### Characterization of the isolated compounds

^1^H NMR spectrum of compound **3** revealed two downfield doublet signals at δ_H_ 6.49 (H-7) and 6.23 (H-6) showing vicinal coupling with coupling constant 8.8 and 8.4 Hz, respectively; two doublets of doublet signals appearing at δ_H_ 5.11 and 5.20 showing trans vicinal coupling (*J* = 7.6, 15.2 Hz) assigned for an exocyclic double bond (H-22 and H-23, respectively). In addition, an oxygenated methine signal at δ_H_ 3.95 and six methyl signals including two singlet signals of 18 and 19 observed at δ_H_ 0.79 and 0.86, respectively, and four doublet signals of 21, 26, 27, and 28 observed at δ_H_ 0.98, 0.78, 0.81 and 0.89, respectively (Yoo et al. 2006; Govindharaj et al. 2019). The ^13^C NMR showed 28 carbon signals including 4 olefinic carbons at δ_C_ 135.5 (C-6), 135.3 (C-22), 132.4 (C-23) and 130.8 (C-7), representing the ergosterol derivative holding the sterol fragment. Furthermore, one oxygenated methine carbon at δ_C_ 66.5 (C-3), and two oxygenated quaternary carbon signals at δ_C_ 79.5 (C-8) and 82.3 (C-5) suggested the presence of peroxide structure. Finally, six methyl signals at δ_C_ 13.0, 18.3, 19.7, 20.1, 20.7, and 17.7 were assigned to carbon atoms 18, 19, 21, 26, 27 and 28, respectively. Accordingly, compound **3** was identified as ergosterol peroxide (Shin et al. 2001).

Positive ESI-Mass analysis of compounds 4, 5 and 6, showed the same molecular weight of 570 ([M-H]^+^ at *m/z* 571), indicating a molecular formula of C_32_H_26_O_10_, and the presence of three isomers with 20 degrees of unsaturation in each case.^1^H and ^13^C NMR spectra of the three compounds showed common characters, including the presence of 32 carbon signals, of which two are methyl and four are methoxyl groups, in addition to six sp^2^ methines, as well as 20 quaternary sp^2^ carbon atoms (including two carbonyl carbon atoms), characteristic for hexacyclic compounds. Furthermore, the presence of two singlet methyl and four methoxyl groups, in addition to two meta-coupled aromatic protons and four singlet aromatic protons, suggested the three compounds to be different dimers of naphtho-γ-pyrones (Akiyama et al. 2003).

The ^1^H NMR spectrum of compound 6 exhibited signals for two singlet methyl groups at δ_H_ 2.47 and 2.54, four methoxyl groups at δ_H_ 3.60, 3.60, 3.79, and 3.99 ppm, together with two *meta*-coupled aromatic protons at δ_H_ 6.18 and 6.43 (d, *J* = 2.4), in addition to four singlet aromatic protons at δ_H_ 6.31, 6.32, 6.97 and 7.02 ppm, respectively. These data suggested that compound **6** to be an asymmetric dimer of flavasperone. Correlations observed in the HMBC spectra allowed the location of the four methoxyl groups (δ_H_ 3.79, δ_C_ 56.2; δ_H_ 3.60, δ_C_ 55.3; δ_H_ 3.60, δ_C_ 61.6 and δ_H_ 3.99, δ_C_ 56.1) at C-8 C-10, C-8 and C-10 (159.6), respectively. The two methyl groups (δ_H_ 2.47, δ_C_ 20.7; δ_H_ 2.54, δ_C_ 20.7) were located at C-2 (167.0) and C-2respectively, as determined by the HMBC correlations. Protons of the former methyl group correlated with C-2 and C-3 (110.7), while protons of the latter group showed correlations with C-2 and C-3 respectively. Based on its spectroscopic properties, and by comparison with reported data compound 6 was identified as the asymmetric dimer of flavasperone linked at C-9 and C-6, Asperpyrone B Akiyama et al. 2003.

Isomerization of one of the angular flavasperone moieties was observed in both compounds 4 and 5. Compound 5 showed an upfield shift of CH_3_-2 (δ_H_ 2.39) and H-3 (δ_H_ 6.02), together with the downfield shift of H-10 (δ_H_ 7.11), indicating that the C-9 linked flavasperone moiety was isomerized to linear rubrofusarin B. Accordingly, compound 5 was identified as Asperpyrone C (Akiyama et al. 2003). In contrast, compound 4 showed an upfield shift of CH_3_-2 (δ_H_ 2.11) and H-3 (δ_H_ 6.00), indicating that C-6 angular flavasperone moiety was isomerized to linear rubrofusarin B. Based on its spectroscopic properties, and by comparison with reported data, compound 4 was identified as a dimer of angular flavasperone and linear rubrofusarin B, Fonsecinone A (Campos et al. 2005).

### Antimicrobial activity

Over the past few decades, the global evolution of antimicrobial resistance (AMR) lead to a progressive decrease in the effectiveness of current drugs used to treat infectious diseases. Discovering novel efficient molecules that could assist in the decline of some of this burden is constantly in demand. Endophytic fungi are considered a promising source of novel bioactive compounds with broad spectrum antimicrobial activities (Caruso et al. 2022).

The EtOAc extracts and some isolated compounds from the endophytic fungus *A. alternata* were reported to have potent antimicrobial (Shaaban et al. 2012; Fernandes et al. 2009), antifungal (Abdou and Dawoud 2020) and antibacterial activities (Hawas et al. 2015; Chatterjee et al. 2019).

*Clostridium perfringens* is the most distributed pathogen in nature causing the most common foodborne illness globally in addition to wound infections like gas gangrene and enterotoxaemia, where toxins produced in the intestine are absorbed and harm distant organs like the brain (Lindström et al. 2011; Gohari et al. 2021).

*Pseudomonas aeruginosa* considers one of the most challenging organisms implicated in a number of infectious diseases (Vo et al. 2022). In 2017, World Health Organization established a list of bacteria with high levels of antibiotic resistance that should deserve priority for the development of new antibiotics, categorizing *Pseudomonas aeruginosa* among the most critical list (WHO, 2017).

Dimeric naphtho-γ-pyrones are reported to have antibacterial activity (Lu et al. 2014). This is in accordance with results obtained in this study, which revealed that compounds 6 and 4 were the most active tested compounds. In addition, He et al. (2016) reported that the angular monomer flavasperone has higher antibacterial activity than its linear isomer rubrofusarin B. This supports our findings, which showed that compound 6, a dimeric naphtho-γ-pyrone of two angular flavasperone monomers with C9/C6` linkage, showed higher antibacterial activity against the two pathogenic bacteria *P. aeruginosa* and *C. perfringens,* with MICs of 0.75 and 1.5 mg/mL, respectively as compared to compound 4, which is a dimer of an angular flavasperone monomer and a planar rubrofusarin B, with MIC of 3 mg/mL against *C. perfringens.* Furthermore, He et al. (2016) studied the antibacterial effect of a group of angular-linear and linear–linear dimeric naphtho-γ-pyrones with different linkage positions; results revealed that C9/C10` was the most active, followed by C7/C10` then C7/C6`. It is worth mentioning that our findings suggest that dimeric naphtho-γ-pyrones with C9/C6` linkage have more antibacterial activity than C9/C10`isomers.

Ergosterol peroxide is reported to have antimicrobial activity, as it effectively inhibited the growth of *Staphylococcus aureus, Escherichia coli* and *Candida albicans* (Tawfik et al. 2017). This supports our findings, which showed that it has significant antibacterial activity.

Molecular Docking results supported the findings of the antibacterial activity study. The predominant factor that contributes to the development of resistance in multidrug-resistant *E. coli* strains is an overexpression of the AcrAB–TolC efflux pump system (Du et al. 2015). The periplasmic protein adaptor AcrA is part of a trimeric complex that preserves AcrB and TolC linked together (Du et al. 2014). Protomers of the homotrimer AcrB can take on three distinct conformations: access, binding, and extrusion. Site-directed mutagenesis and real-time efflux investigations have verified the mechanism by which these pumps expel the medication, demonstrating that the proton-motive force drives substrate transport across this system (Blair et al. 2015). Some antibiotics and numerous inhibitors, Efflux pump inhibitors (EPIs) have been co-crystallized with the AcrB pump and were used to study the binding interactions within the substrate binding site (Eicher et al. 2012a). Clustal Omega (Thompson et al. 1994) was used to align the protein sequence (PDB code: 5ENR) with the AcrB crystal structure (PDB code: 4DX5) (Eicher et al. 2012b). Solving the crystal structure (PDB code: 5ENR) has shown that pyranopyridines (MBX3135) (Opperman et al. 2014; Nguyen et al. 2015) block the way that substrates can get into AcrB’s deep binding pocket. They attach together in a cage packed of phenylalanine (phe136, phe178, phe610, phe615, and phe628), which expands out from the dimer's deep binding pocket to create a net of hydrophobic connections that effectively blocks the trimer’s ability to rotate functionally. The production of a complex hydrogen bond network between proteins and water contributes for the increased efficacy of inhibitors (Lamut et al. 2019). Comparison of the structures of MBX3135 and other powerful EPIs reveals that they all feature at least two hydrophobic ring systems that can interact with hydrophobic amino acid residues in the substrate binding site. Re-docking the co-crystallized ligand (MBX3135) into AcrB binding sites validated the MOE molecular docking protocol. The original poses generated from PDB were retrieved with root mean square deviation (RMSD) values in the range of, 0.30 Å and binding energy scores (S score) of − 11.5079 kcal/mol (Table 4).

The docking study of compounds 6, 4, 1 and 3 showed that all compounds occupied the deep binding pocket within a phenylalanine‐rich cage and superimposed the co-crystallized ligand with RMSD values 0.51, 0.46, 1.40 and 0.84 Å and binding energy scores of − 9.4661, − 8.8413, − 7.68 and − 5.99 kcal/mol respectively.

The pyridine ring of MBX3135 stacks with the ring of phe628 on one side and interacts with Val 137 on the other, while the morpholine ring was anchoring at the pockets through *π-π* stacking with side-chain phenyl moiety of phe615, and the phenylethylthio group is loosely located in a hydrophobic pocket surrounded by phe178, Ile277, Ala279, and Val612. Meanwhile, water molecules serve hydrogen bonding between the nitrogen of acrylamide and Gln151 to enhance the binding affinity (Fig. 4a, d).

The most active antibacterial compounds 6 and 4 bind with high affinity in the phenylalanine-rich cage (phe136, phe178, phe610, phe615, and phe628) and they are surrounded with another hydrophobic residue Val 139, Ile277, Ala279. Pro 326, Val571, and Val612 (Additional file 1).

The central, Benzo[*h*]chromene-4-one ring moiety of compound 6 is oriented parallel to the ph628 aromatic side chain with a distance of ∼4.3 Å, resulting in an extensive π–π stacking interaction. Similarly, the phenyl ring of the other benzo[h]chromene experienced a hydrophobic interaction with phe178 at a distance of ∼2.8 Å. Interestingly, water molecule promotes strengthening the binding through H-bonding between 5-hydroxy substituent and Gln151 at a distance of 1.86 and 1.67 Å respectively (Fig. 4b, e).

In case of compound 4, benzo[*h*]chromen-4-one scaffold participated in a hydrophobic interaction with Phe178, in addition to a hydrophobic interaction of C*α* hydrogen of Phe617 with benzo[*g*]chromen-4’-one moiety (Fig. 4c, f).

### Molecular docking into ATP-binding pocket of DNA gyrase B

DNA gyrase is type II topoisomerase (Maxwell and Lawson 2003; Collin et al. 2011) and is composed of two GyrA and GyrB subunits, where GyrA participates in the cleavage and recombination of DNA, while the other part GyrB has ATPase activity, providing the essential energy needed for DNA cleavage and recombination.

In the present investigation, the proposed molecular docking algorithm was initially validated by re-docking of the co-crystallized ligand (6G9) to the active site DNA-gyraseB (pdb ID: 5L3J). The validation results of 6G9 revealed a score energy of -6.0096 kcal/mol and RMSD 0.95 Å. The docking poses achieved interactions with the key amino acids in the active site through hydrogen bonds Val71, Asp73, and Arg136 (Fig. 5a, d).

The obtained scoring results of compounds 6, 4, 1 and 3 showed that they occupied the ATP binding site DNA gyrase B with good binding affinity. They bind with docking scores of − 6.09, − 5.93, − 5.36 and − 5.10 kcal/mol, with RMSD scores of 0.71, 1.52, 1.21 and 1.70, respectively.

The molecular docking results of the most active compound 6 showed that the hydroxy group of Benzo[*h*]chromene-4-one moiety is oriented toward Asp 72 amino acid and the polar hydrogen atom (OH) is involved in hydrogen bond with a distance of 2.17 Å. This might be attributed to its higher ATPase potency against DNA gyrase (Fig. 5b, e). In contrast, compound 4 (Fig. 5c, f) lack the ability to form hydrogen bond interaction with Asp 72 that might be attributed to the linear arrangement of benzochromene-4-one but (5-OH) of Benzo[*h*]chromene-4-one moiety is close to Glu50 and Gly77 with distances 2.61 and 2.87 Å, respectively (Table 4).

### Antiproliferative effect of isolated endophytic metabolites (1–6) against different prostate cancer cells

Prostate cancer is one of the highest prevalent cancers in 2020, being the second diagnosed and the fifth leading cause of death in men globally (Sung et al. 2021). Aiming to find promising lead compounds against prostate cancer, antiproliferative activity study was performed against different cell lines including DU-145, PC-3, PC-3 M, 22Rv1 and CWR-R1ca, using MTT assay. These cell lines represent a panel with different genotypes and phenotypes characteristics that widen the scope of the screening campaign. For instance, DU-145 cells are lacking the expression of androgen receptor (AR), and are thus unresponsive to androgen stimulation. It is also tumorigenic in nude mouse models and metastatic (originally isolated from a brain metastatic lesion) (Chlenski et al. 2001; Stone et al. 1978). Furthermore, PC-3 and PC-3 M cells are other androgen-independent cell lines with AR-expression negative and have the mesenchymal phenotype with excellent cell migration in various in vitro motility assays (Chlenski et al. 2001; Siddique et al. 2022). CWR-R1ca is an androgen-sensitive cell line that expresses the androgen receptor and is known to be highly aggressive, metastatic, and castration-resistant phenotype of human prostate adenocarcinoma (Siddique et al. 2022; Shourideh et al. 2016). 22Rv1 is an androgen-responsive cell line and has become a valuable model to study androgen receptor function, in addition to the efficacy of current drugs and designing novel anti-androgen receptor therapies (Sampson et al. 2013).

Dimeric naphtho-γ-pyrones are reported to have strong cytotoxic and antitumor activities (Lu et al. 2014). Antonov et al. (2019) evaluated the cytotoxic activity and inhibition of colony formation of different naphtho-γ-pyrones on human drug-resistant prostate cancer 22Rv1 cells. Among the tested compounds were the angular-angular dimer asperpyrone B and the angular-linear dimer fonsecinone A; results revealed that the latter was by far the more cytotoxic compound, with IC_50_ value 13.13 μM, and showed strong inhibition of colony formation with an inhibition percent of 88.71%. Furthermore, asperpyrone B showed weak cytotoxic activity (IC_50_ = 95.54 μM) and did not exhibit any inhibition of colony formation. This is in accordance with the results of the current study, which revealed that the angular-linear compound 4 was the most active antiproliferative, followed by the other tested angular-linear compound 5, while the least active dimeric naphtho-γ-pyrones was the angular-angular dimer compound 6. Therefore, from previous studies, as well as the results of our study, it could be concluded that angular-linear dimeric naphtho-γ-pyrones could be promising lead compounds for antiprostatic cancer drug development. Moreover, it is worth mentioning that compound 4 showed the lowest IC_50_ values against the two androgen-sensitive cell lines CWR-R1ca and 22Rv1, with IC_50_ values 13.3 and 17.1 μM, respectively. http://www.blast.ncbi.nlm.nih.gov/Blasthttps://www.who.int/news-room/fact-sheets/detail/cancer

## Supplementary Information

**Additional file 1:** **Figure S1.** ^1^H NMR spectrum (400 MHz, CDCl_3_) of compound 1. **Figure S2.** ^13^C NMR spectrum (100 MHz, CDCl_3_) of compound 1. **Figure S3.** HMBC spectrum of compound 1. **Figure S4.** 1H NMR spectrum (400 MHz, CDCl_3_) of compound 2. **Figure S5.** ^13^C NMR spectrum (100 MHz, CDCl_3_) of compound 2. **Figure S6.** Positive ESI-MS spectrum of compound 3. **Figure S7.** ^1^H NMR spectrum (400 MHz, CDCl_3_) of compound 3. **Figure S8.** ^13^C NMR spectrum (100 MHz, CDCl_3_) of compound 3. **Figure S9.** HMQC spectrum of compound 3. **Figure S10.** HMBC spectrum of compound 3. **Figure S11.** Positive ESI-MS spectrum of compound 4. **Figure S12.** ^1^H NMR spectrum (400 MHz, CDCl_3_) of compound 4. **Figure S13.** ^13^C NMR spectrum (100 MHz, CDCl_3_) of compound 4. **Figure S14.** HMQC spectrum of compound 4. **Figure S15.** HMBC spectrum of compound 4. **Figure S16.** Positive ESI-MS spectrum of compound 5. **Figure S17.** ^1^H NMR spectrum (400 MHz, CDCl_3_) of compound 5. **Figure S18. **^13^C NMR spectrum (100 MHz, CDCl_3_) of compound 5. **Figure S19.** HMQC spectrum of compound 5. **Figure S20.** HMBC spectrum of compound 5. **Figure S21.** Positive ESI-MS spectrum of compound 6. **Figure S22.** ^1^H NMR spectrum (400 MHz, CDCl_3_) of compound 6. **Figure S23.** ^13^C NMR spectrum (100 MHz, CDCl_3_) of compound 6. **Figure S24.** HMBC spectrum of compound 6. **Figure S25.** Inhibitory effect of different concentrations (1, 0.75 and 0.5 mg/ml) of ETOAC extract against reference strains. (A) = Escherichia coli ATCC 8739 (E.C), (B) = Pseudomonas aeruginosa ATCC 9027 (Ps.), (C) = Klebsiella pneumonia ATCC 700603 (K), (D) = Salmonella enterica ATCC 14028 (SL.), (E)= Listeria monocytogenes ATCC 7644 (Ls.), (F) = Clostridium perfringens ATCC 13124 (CL.), (G) Staphylococcus aureus ATCC 25923 (S1), (H) = Streptococcus faecalis ATCC 8043 (S2), (I)= Candida albicans ATCC 10231 (C), and (J)= Aspergillus niger ATCC 6275 (Asp.). **Figure S26.** Inhibitory effect of different concentrations (1, 0.75 and 0.5 mg/ml) of pure compound 1 against reference strains. (A) = Escherichia coli ATCC 8739 (E.C), (B) = Pseudomonas aeruginosa ATCC 9027 (Ps.), (C) = Klebsiella pneumonia ATCC 700603 (K), (D) = Salmonella enterica ATCC 14028 (SL.), (E)= Listeria monocytogenes ATCC 7644 (Ls.), (F) = Clostridium perfringens ATCC 13124 (CL.), (G) Staphylococcus aureus ATCC 25923 (S1), (H) = Streptococcus faecalis ATCC 8043 (S2), (I)= Candida albicans ATCC 10231 (C), and (J)= Aspergillus niger ATCC 6275 (Asp.). **Figure S27.** Inhibitory effect of different concentrations (1, 0.75 and 0.5 mg/ml) of pure compound 3 against reference strains. (A) = Escherichia coli ATCC 8739 (E.C), (B) = Pseudomonas aeruginosa ATCC 9027 (Ps.), (C) = Klebsiella pneumonia ATCC 700603 (K), (D) = Salmonella enterica ATCC 14028 (SL.), (E)= Listeria monocytogenes ATCC 7644 (Ls.), (F) = Clostridium perfringens ATCC 13124 (CL.), (G) Staphylococcus aureus ATCC 25923 (S1), (H) = Streptococcus faecalis ATCC 8043 (S2), (I)= Candida albicans ATCC 10231 (C), and (J)= Aspergillus niger ATCC 6275 (Asp.). **Figure S28.** Inhibitory effect of different concentrations (1, 0.75 and 0.5 mg/ml) of pure compound 4 against reference strains. (A) = Escherichia coli ATCC 8739 (E.C), (B) = Pseudomonas aeruginosa ATCC 9027 (Ps.), (C) = Klebsiella pneumonia ATCC 700603 (K), (D) = Salmonella enterica ATCC 14028 (SL.), (E)= Listeria monocytogenes ATCC 7644 (Ls.), (F) = Clostridium perfringens ATCC 13124 (CL.), (G) Staphylococcus aureus ATCC 25923 (S1), (H) = Streptococcus faecalis ATCC 8043 (S2), (I)= Candida albicans ATCC 10231 (C), and (J)= Aspergillus niger ATCC 6275 (Asp.). **Figure S29.** Inhibitory effect of different concentrations (1, 0.75 and 0.5 mg/ml) of pure compound 6 against reference strains. (A) = Escherichia coli ATCC 8739 (E.C), (B) = Pseudomonas aeruginosa ATCC 9027 (Ps.), (C) = Klebsiella pneumonia ATCC 700603 (K), (D) = Salmonella enterica ATCC 14028 (SL.), (E)= Listeria monocytogenes ATCC 7644 (Ls.), (F) = Clostridium perfringens ATCC 13124 (CL.), (G) Staphylococcus aureus ATCC 25923 (S1), (H) = Streptococcus faecalis ATCC 8043 (S2), (I)= Candida albicans ATCC 10231 (C), and (J)= Aspergillus niger ATCC 6275 (Asp.). **Figure S30.** Concentration-response curve of isolated metabolites (1-3) on the proliferation of different prostate cancer cell lines. Bar graphs represent mean cell proliferation (±SD) and indicated concentrations. A) compound 1; B) compound 2; C) compound 3. **Figure S31.** Concentration-response curve of isolated metabolites (4-6) on the proliferation of different prostate cancer cell lines. Bar graphs represent mean cell proliferation (±SD) and indicated concentrations. A) compound 4; B) compound 5; C) compound 6.

## Data Availability

The datasets generated during and/or analysed during the current study are available from the corresponding author on reasonable request.
