# Supplementary material for: Antimicrobial, antiproliferative activities and molecular docking of metabolites from Alternaria alternata
Source: AMB Express. 2023 Jul 6;13:68. doi: 10.1186/s13568-023-01568-1 (PMC10326215; doi:10.1186/s13568-023-01568-1)
Supplement: Supplementary file 1 — Additional file 1: Figure S1. 1H NMR spectrum (400 MHz, CDCl3) of compound 1. Figure S2. 13C NMR spectrum (100 MHz, CDCl3) of compound 1. Figure S3. HMBC spectrum of compound 1. Figure S4. 1H NMR spectrum (400 MHz, CDCl3) of compound 2. Figure S5. 13C NMR spectrum (100 MHz, CDCl3) of compound 2. Figure S6. Positive ESI-MS spectrum of compound 3. Figure S7. 1H NMR spectrum (400 MHz, CDCl3) of compound 3. Figure S8. 13C NMR spectrum (100 MHz, CDCl3) of compound 3. Figure S9. HMQC spectrum of compound 3. Figure S10. HMBC spectrum of compound 3. Figure S11. Positive ESI-MS spectrum of compound 4. Figure S12. 1H NMR spectrum (400 MHz, CDCl3) of compound 4. Figure S13. 13C NMR spectrum (100 MHz, CDCl3) of compound 4. Figure S14. HMQC spectrum of compound 4. Figure S15. HMBC spectrum of compound 4. Figure S16. Positive ESI-MS spectrum of compound 5. Figure S17. 1H NMR spectrum (400 MHz, CDCl3) of compound 5. Figure S18. 13C NMR spectrum (100 MHz, CDCl3) of compound 5. Figure S19. HMQC spectrum of compound 5. Figure S20. HMBC spectrum of compound 5. Figure S21. Positive ESI-MS spectrum of compound 6. Figure S22. 1H NMR spectrum (400 MHz, CDCl3) of compound 6. Figure S23. 13C NMR spectrum (100 MHz, CDCl3) of compound 6. Figure S24. HMBC spectrum of compound 6. Figure S25. Inhibitory effect of different concentrations (1, 0.75 and 0.5 mg/ml) of ETOAC extract against reference strains. (A) = Escherichia coli ATCC 8739 (E.C), (B) = Pseudomonas aeruginosa ATCC 9027 (Ps.), (C) = Klebsiella pneumonia ATCC 700603 (K), (D) = Salmonella enterica ATCC 14028 (SL.), (E)= Listeria monocytogenes ATCC 7644 (Ls.), (F) = Clostridium perfringens ATCC 13124 (CL.), (G) Staphylococcus aureus ATCC 25923 (S1), (H) = Streptococcus faecalis ATCC 8043 (S2), (I)= Candida albicans ATCC 10231 (C), and (J)= Aspergillus niger ATCC 6275 (Asp.). Figure S26. Inhibitory effect of different concentrations (1, 0.75 and 0.5 mg/ml) of pure compound 1 against reference strains. (A) = Escherichia coli ATCC 8739 (E. [file 13568_2023_1568_MOESM1_ESM.docx]

**Additional file**

Journal: **AMB Express**

TITLE:  **Antimicrobial, Antiproliferative Activities and Molecular Docking of Metabolites from *Alternaria alternata***

Authors: **Heba T. Khazaal^a^; Mohamed T. Khazaal^b^; Ahmed S. Abdel-Razek^c^; Ahmed A. Hamed^c^; Hassan Y. Ebrahim^a^; Reham R. Ibrahim^a^; Mokhtar Bishr^d^; Yara E. Mansour^e^; Rabab A. El Dib^a^; Hesham S. M. Soliman^a, f *^**

*^a^Department of Pharmacognosy, Faculty of Pharmacy, Helwan University, Ain-Helwan, Cairo 11795, Egypt.*

*^b^Botany and Microbiology Department, Faculty of Science, Helwan University, Cairo 11795, Egypt.*

*^c^Microbial Chemistry Department, National Research Center, 33 El-Buhouth Street, Giza 12622, Egypt.*

*^d^Plant General Manager and Technical Director of the Arab Company for Pharmaceuticals and Medicinal, Plants, Cairo, Egypt.*

*^e^Pharmaceutical Organic Chemistry Department, Faculty of Pharmacy, Helwan University, Ain-Helwan, Cairo 11795, Egypt.*

*^f^PharmD program, Egypt-Japan University of Science and Technology (E-JUST), New Borg El-Arab City, 21934 Alexandria, Egypt.*

*** Corresponding Author:**

Prof. Hesham Soliman
e-mail: hesham.soliman@ejust.edu.eg
PharmD Program
Egypt-Japan University of Science and Technology (E-JUST),
New Borg El-Arab City, 21934 Alexandria, Egypt.


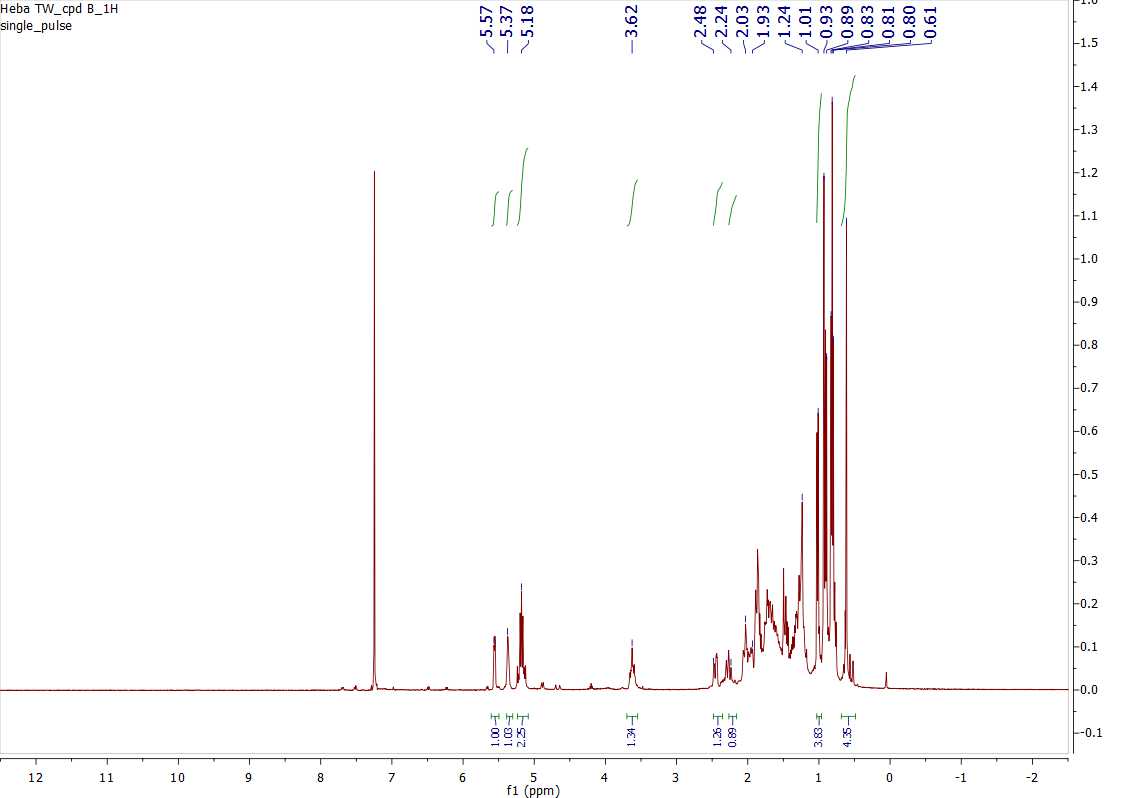
**Figure S1.** ^1^H NMR spectrum (400 MHz, CDCl_3_) of compound 1

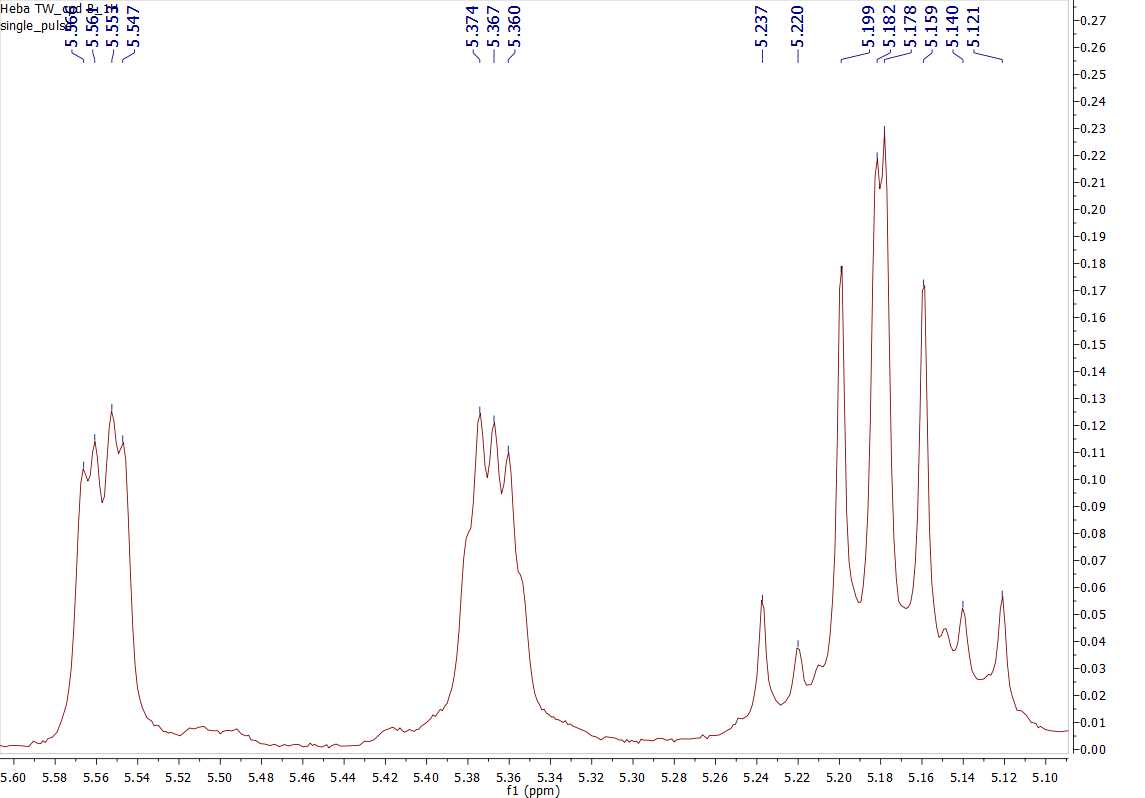


**Figure S2.** ^13^C NMR spectrum (100 MHz, CDCl3) of compound 1


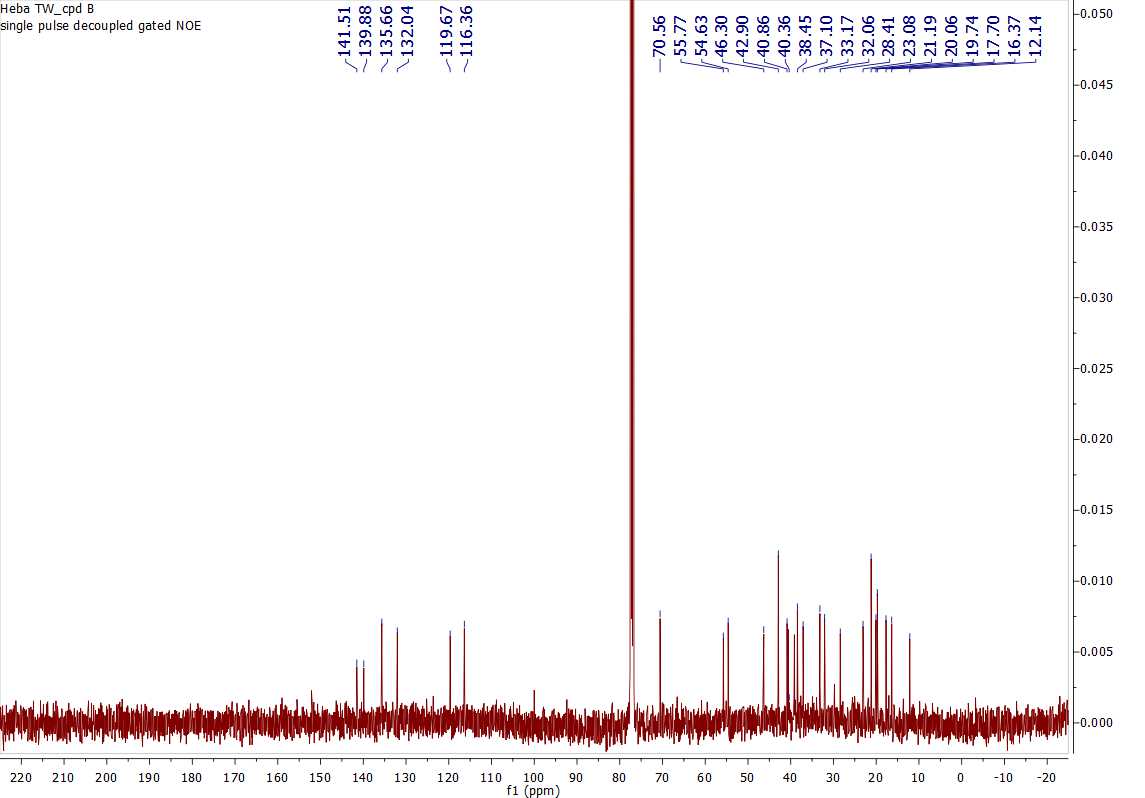

**Figure S3.** HMBC spectrum of compound 1
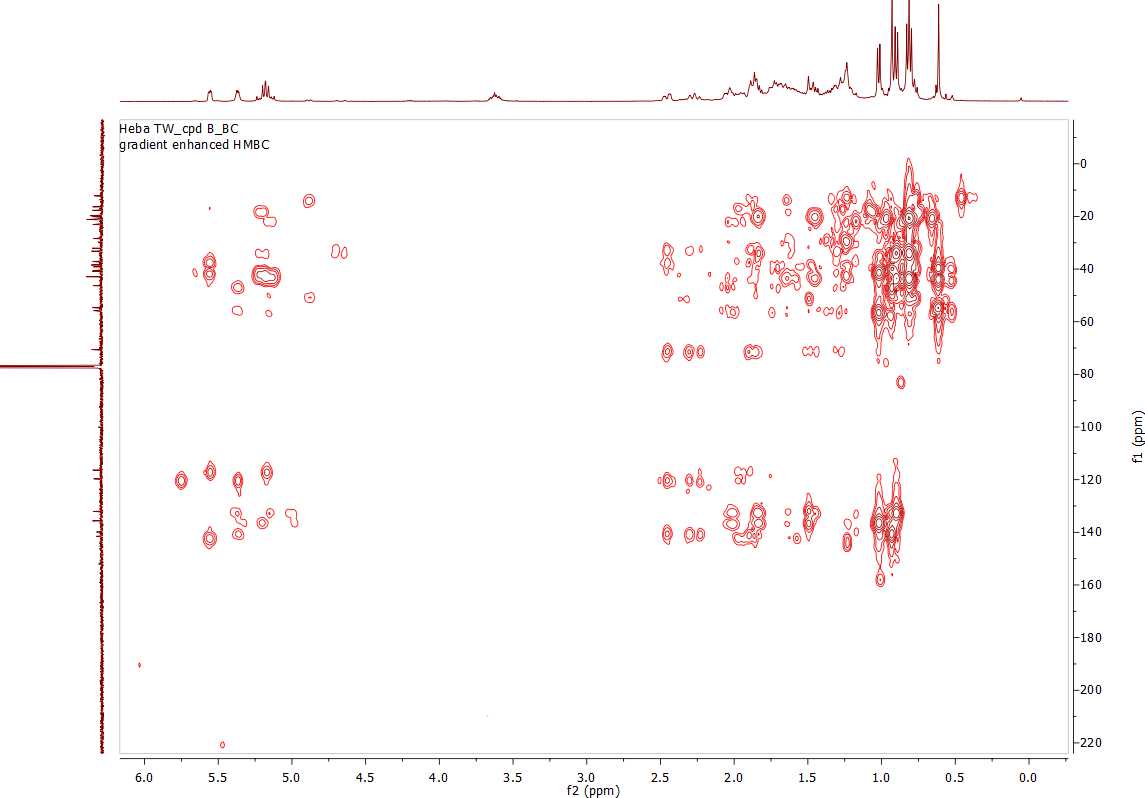

**Figure S4.** ^1^H NMR spectrum (400 MHz, CDCl_3_) of compound 2


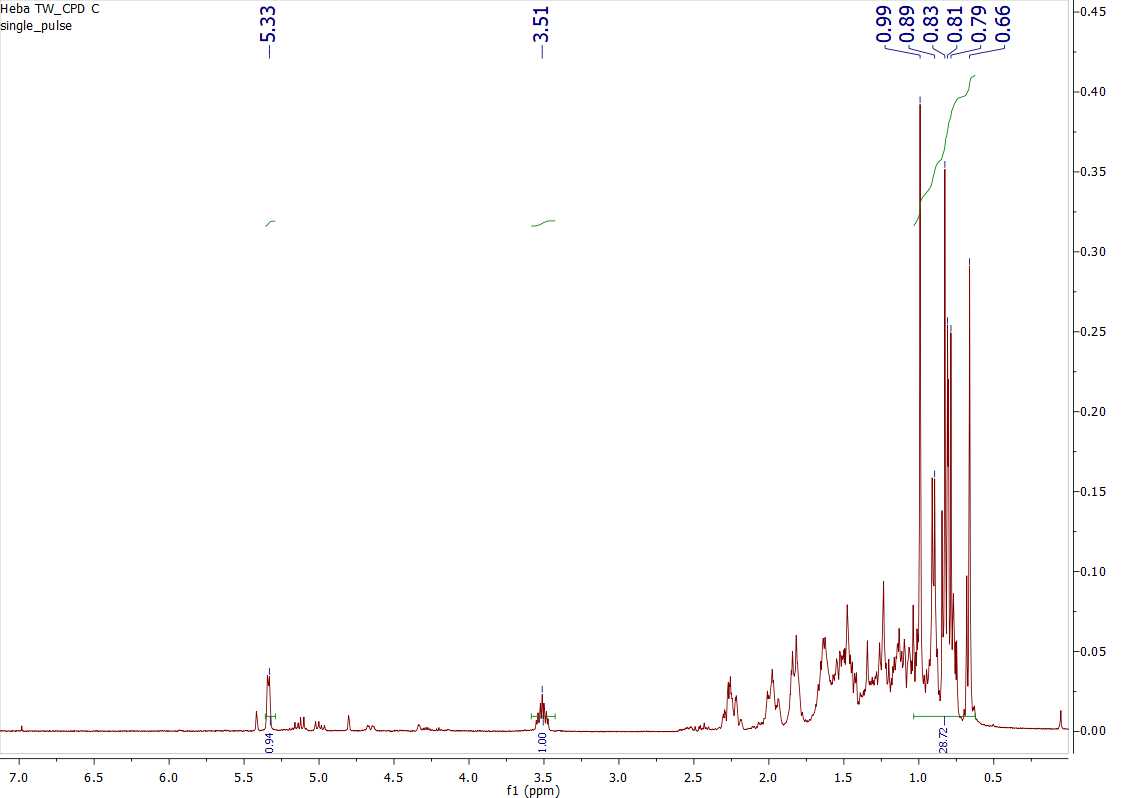

**Figure S5.** ^1^H NMR spectrum (400 MHz, CDCl_3_) of compound 2


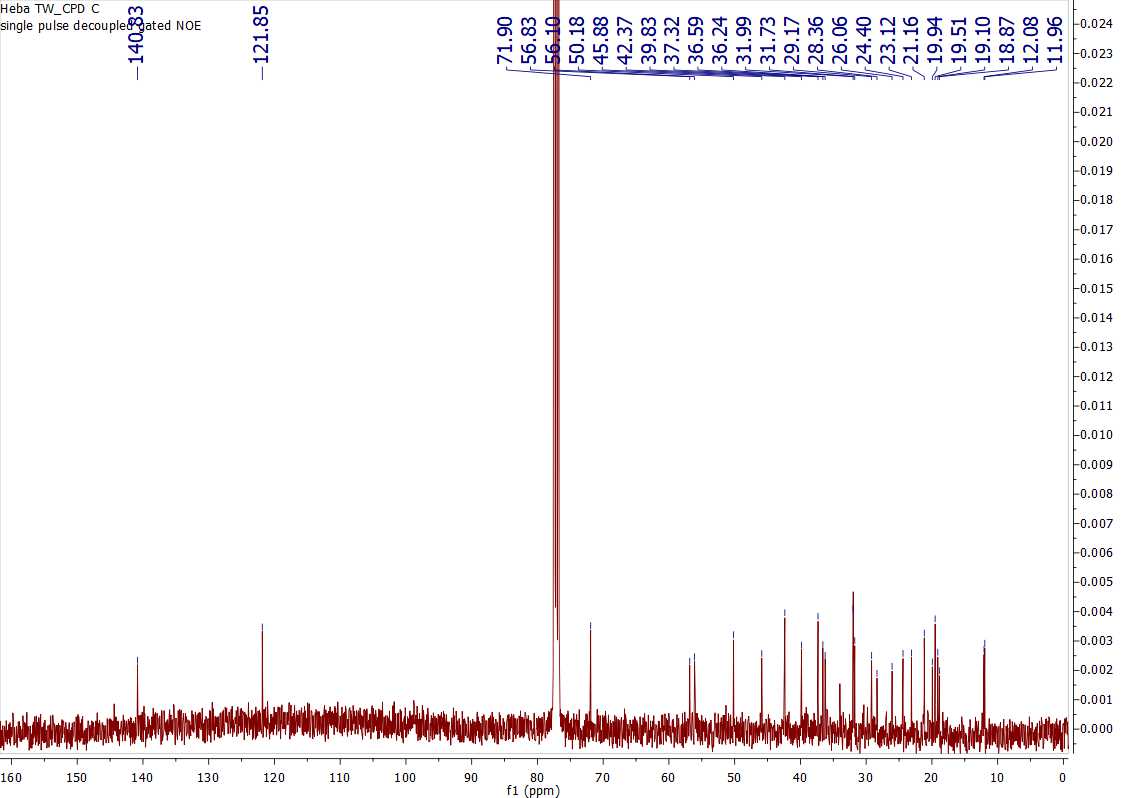

13C NMR spectrum (100 MHz, CDCl3) of compound 2

**Figure S6.** Positive ESI-MS spectrum of compound 3


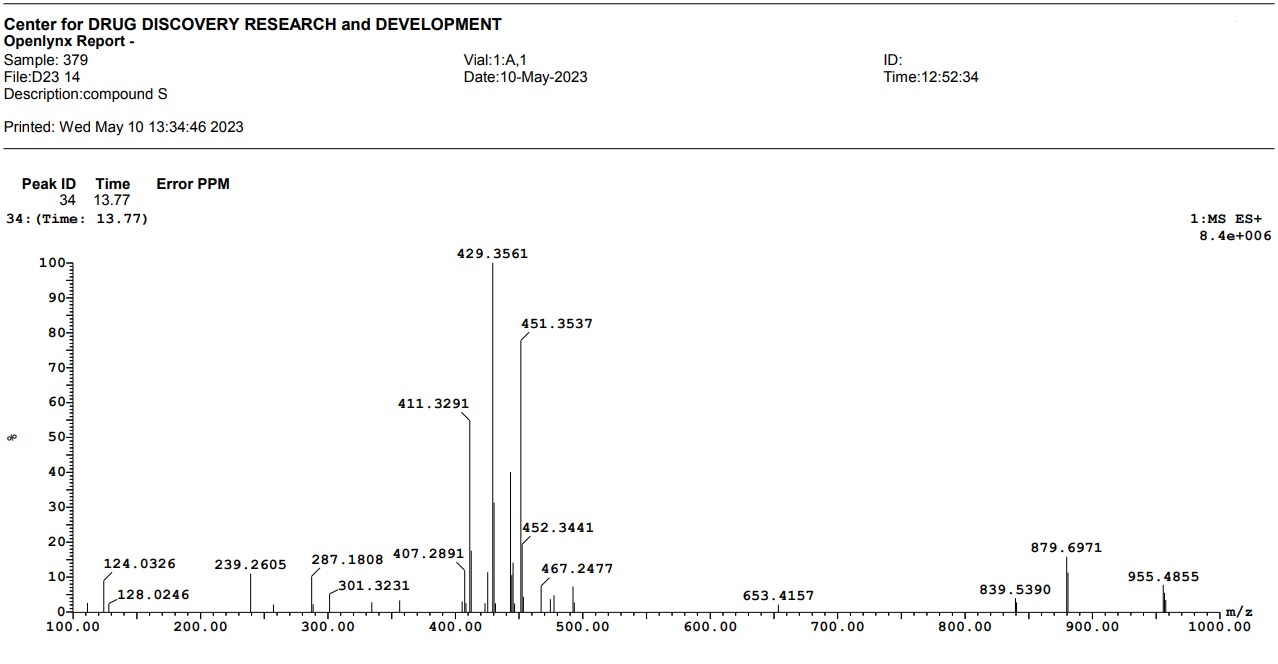


**Figure S7.** ^1^H NMR spectrum (400 MHz, CDCl_3_) of compound 3
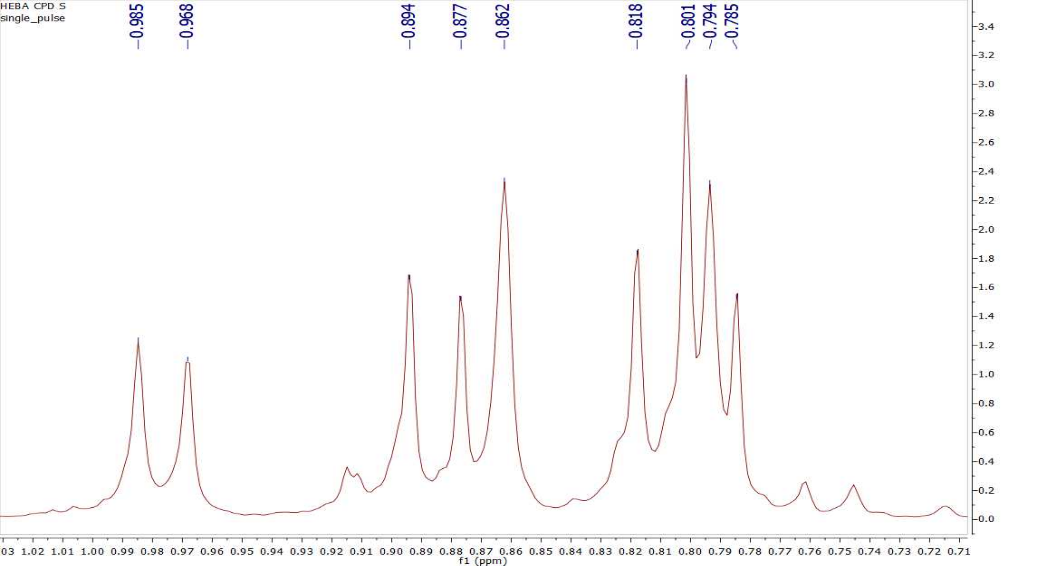


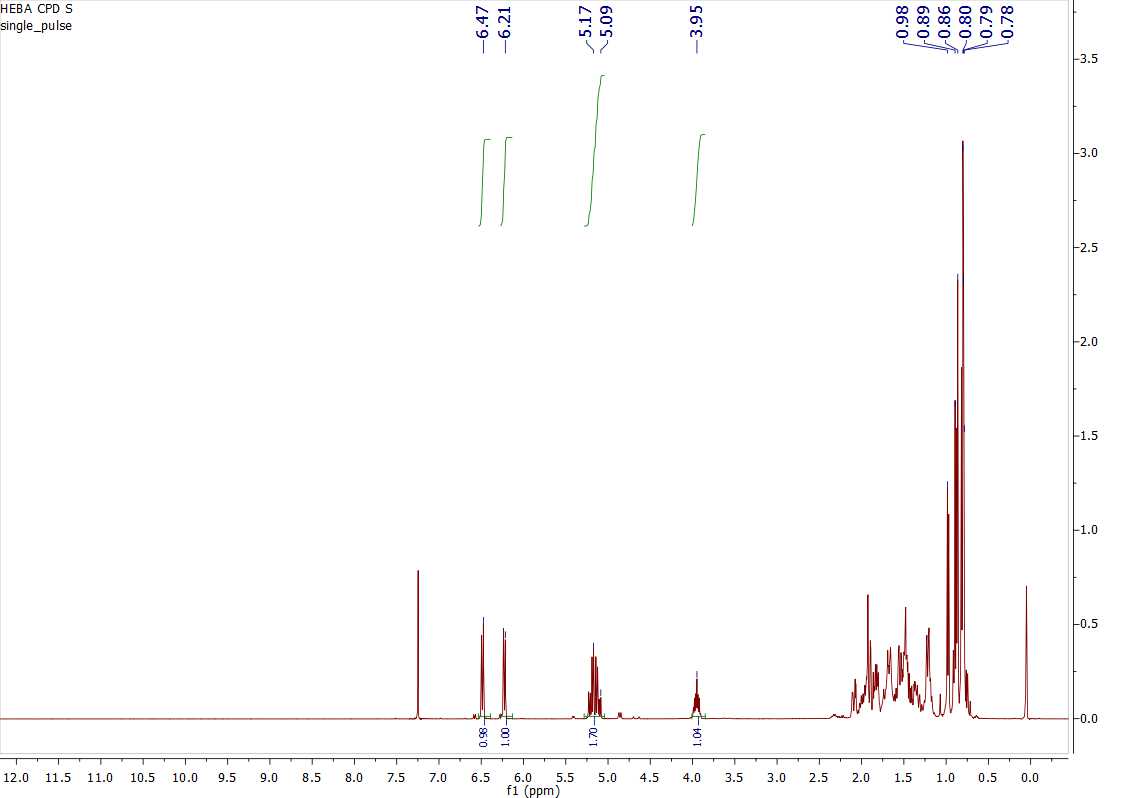


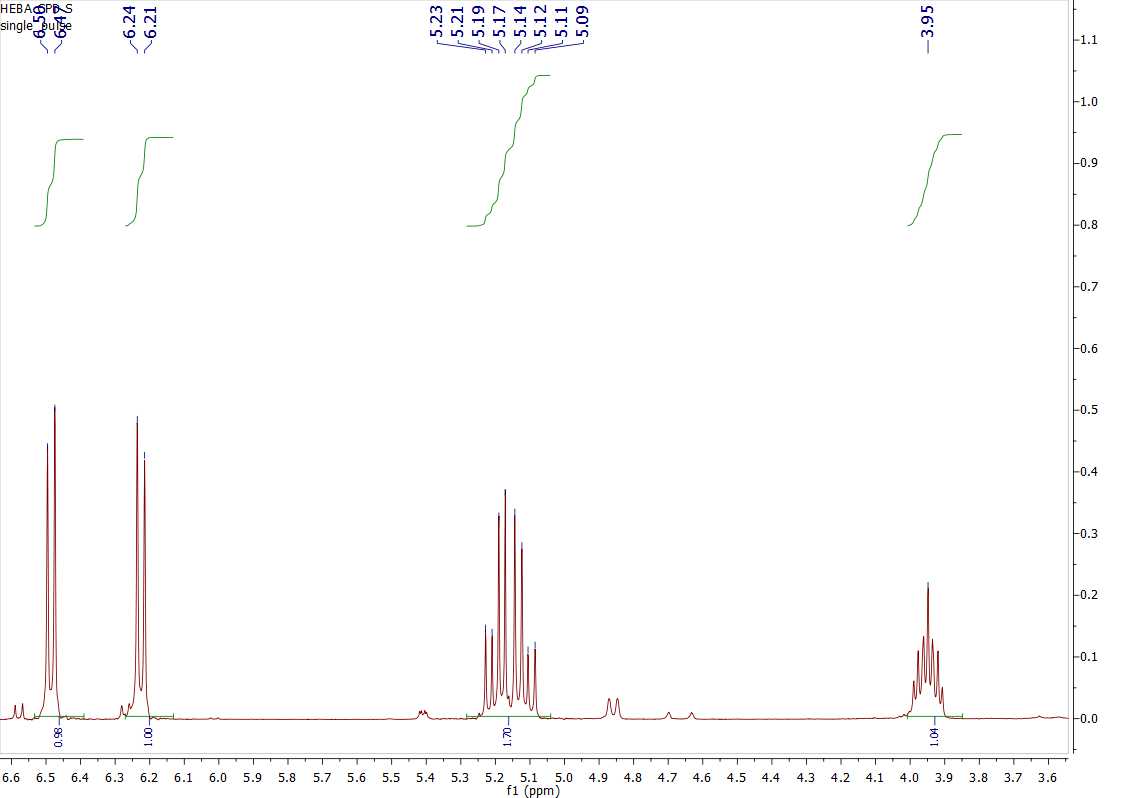


**Figure S8.**
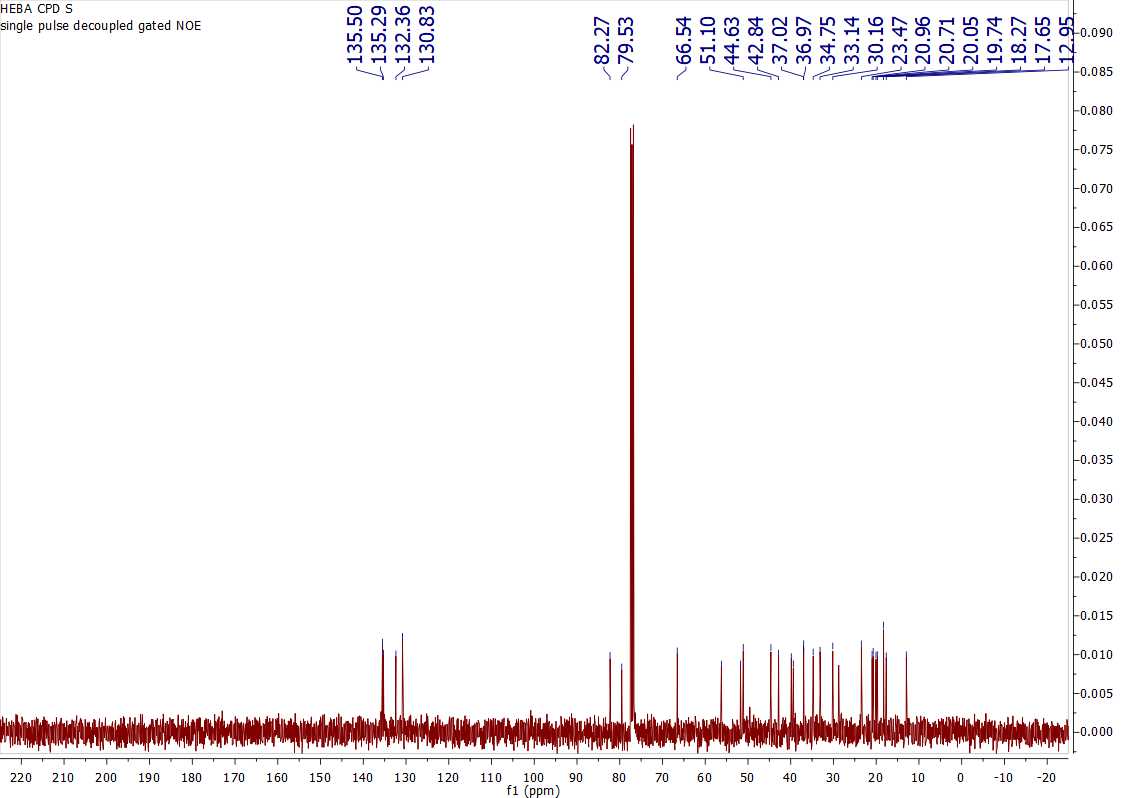
 ^13^C NMR spectrum (100 MHz, CDCl3) of compound 3

**Figure S9.** HMQC spectrum of compound 3


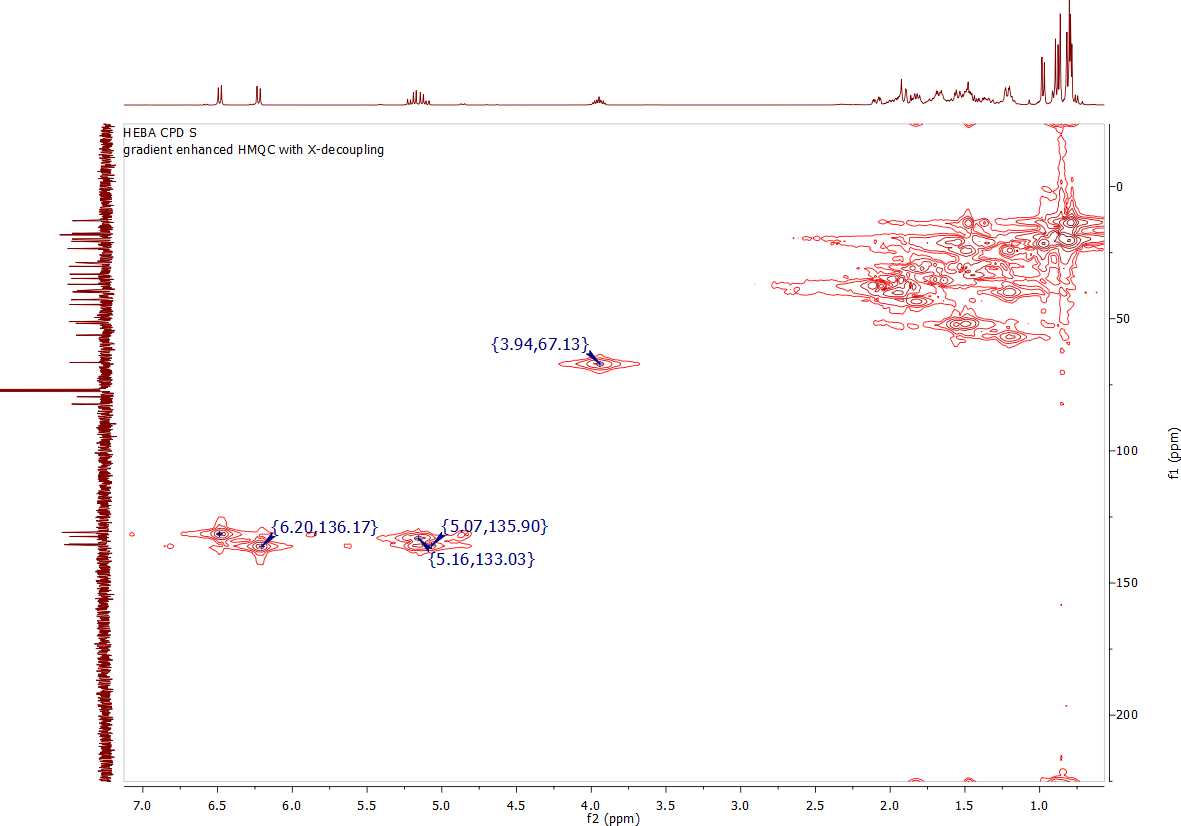

**Figure S10.** HMBC spectrum of compound 3


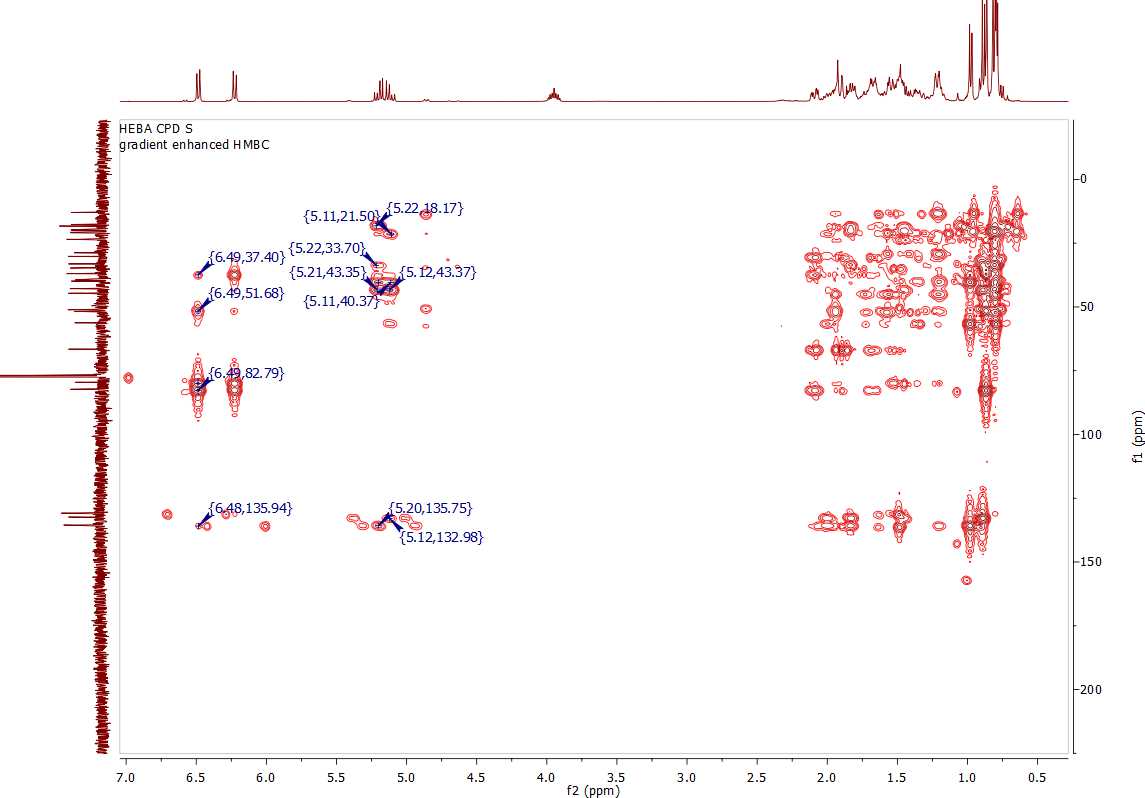

**Figure S11.** Positive ESI-MS spectrum of compound 4


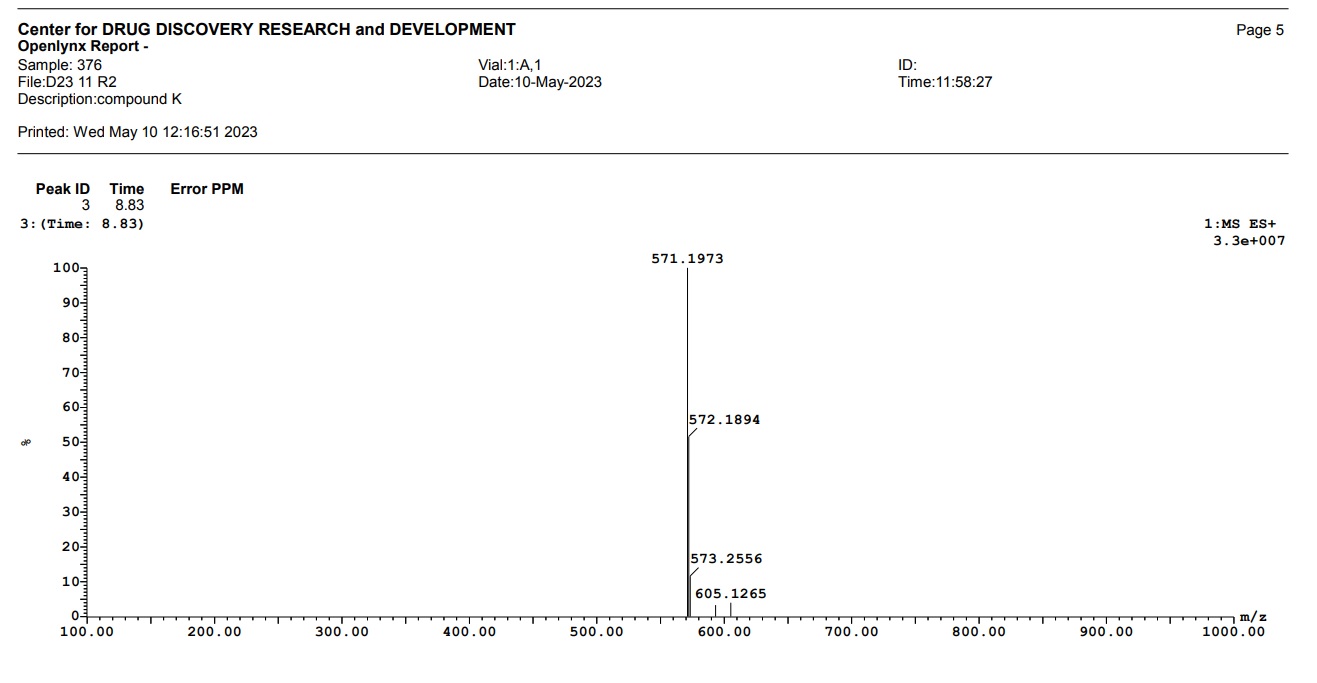


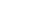


**Figure S12.** ^1^H NMR spectrum (400 MHz, CDCl_3_) of compound 4
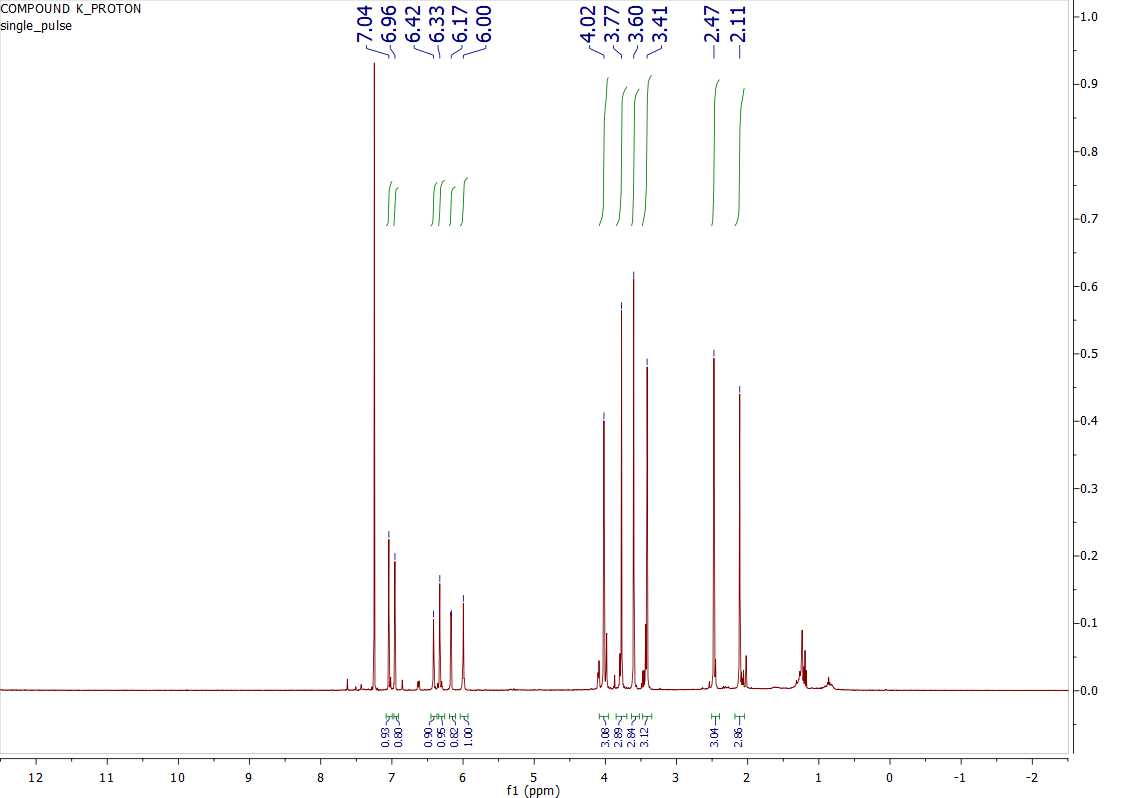

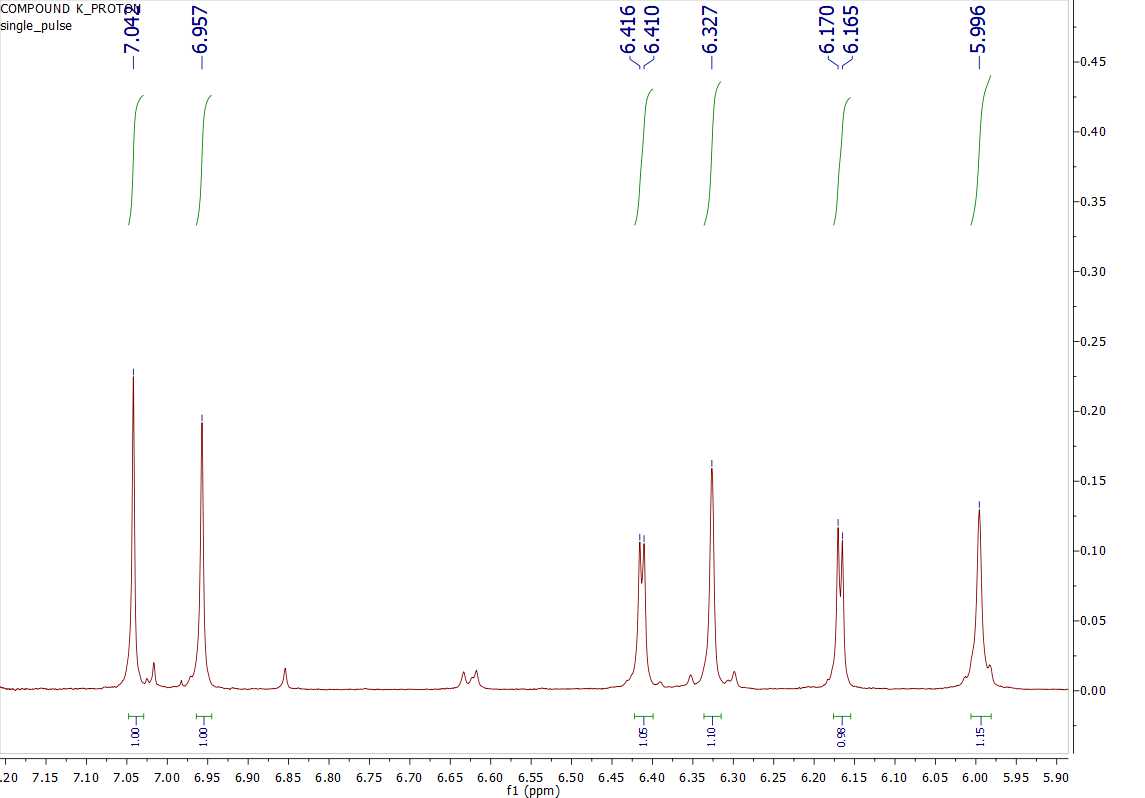


**Figure S13.** 13C NMR spectrum (100 MHz, CDCl3) of compound 4


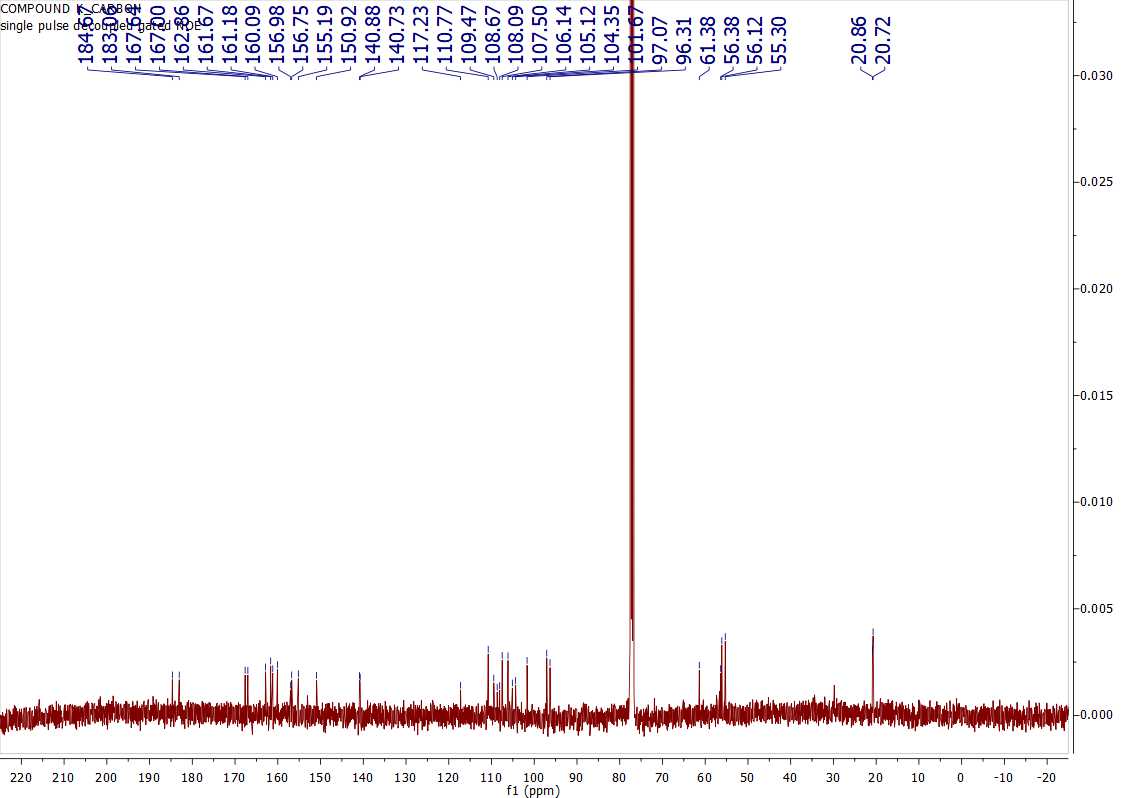

**Figure S1**
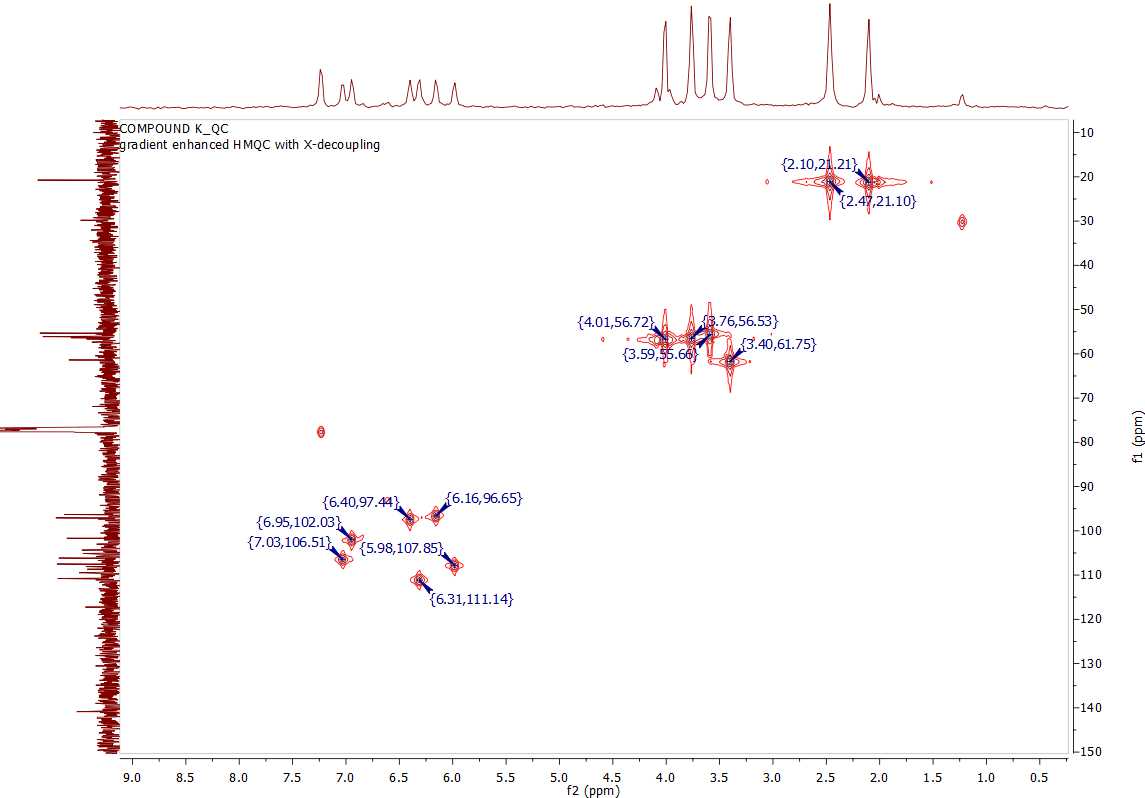
**4.** HMQC spectrum of compound 4

 **Figure S15**. HMBC spectrum of compound 4


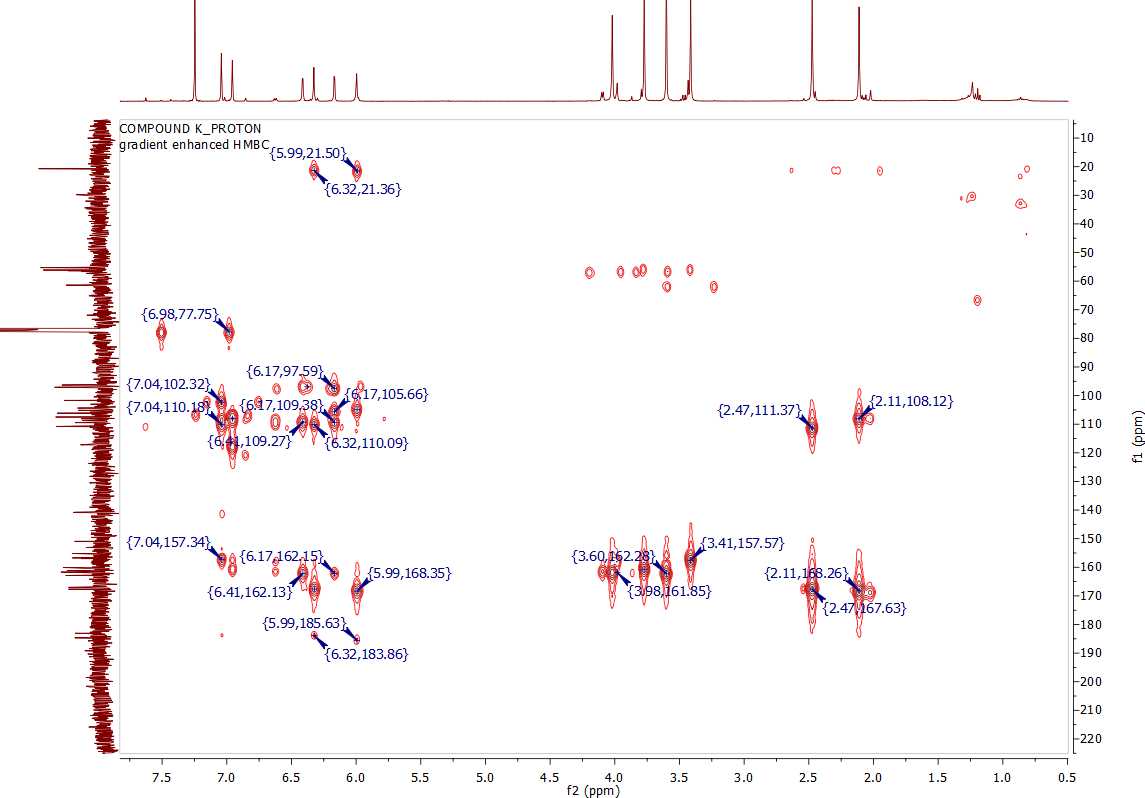

**Figure S1**
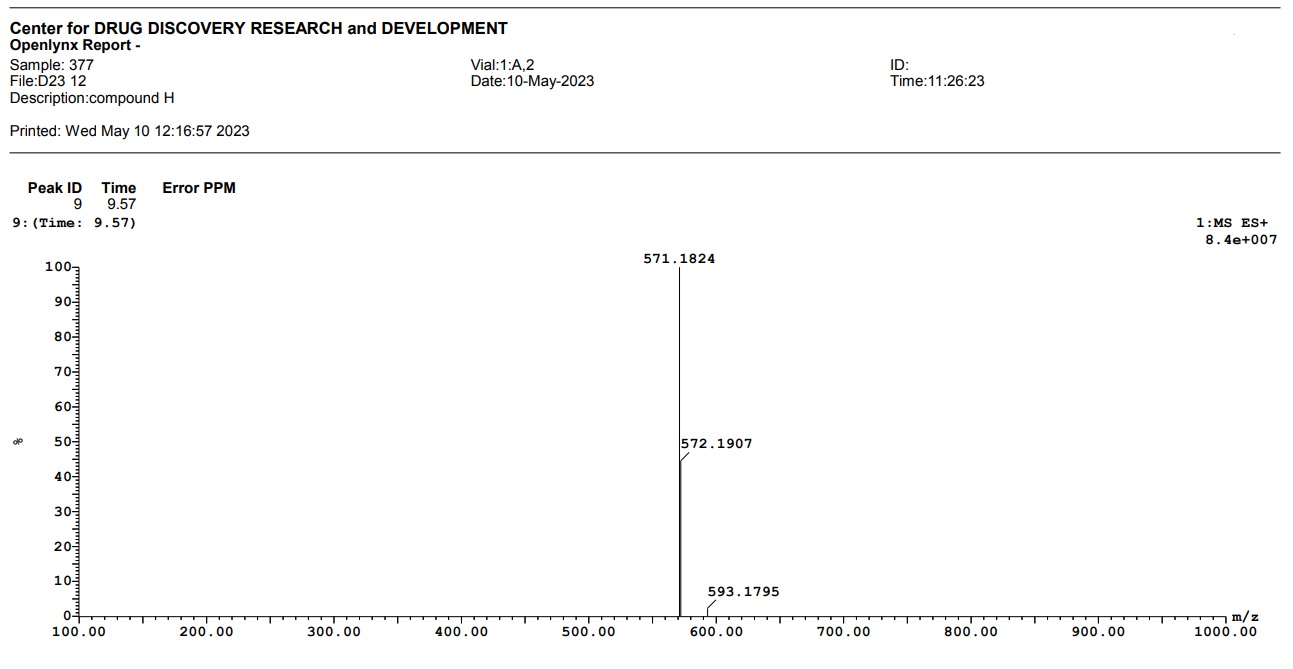
**6.** Positive ESI-MS spectrum of compound 5


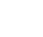


**Figure S17**. ^1^H NMR spectrum (400 MHz, CDCl_3_) of compound 5


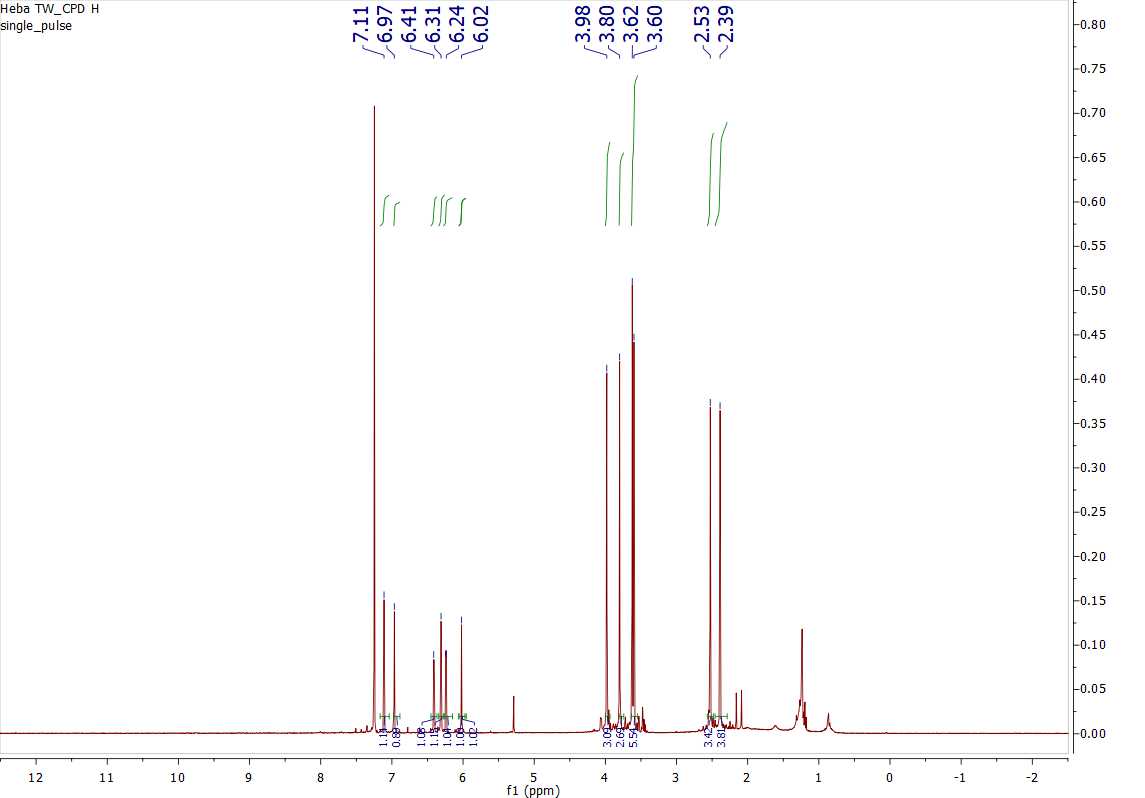

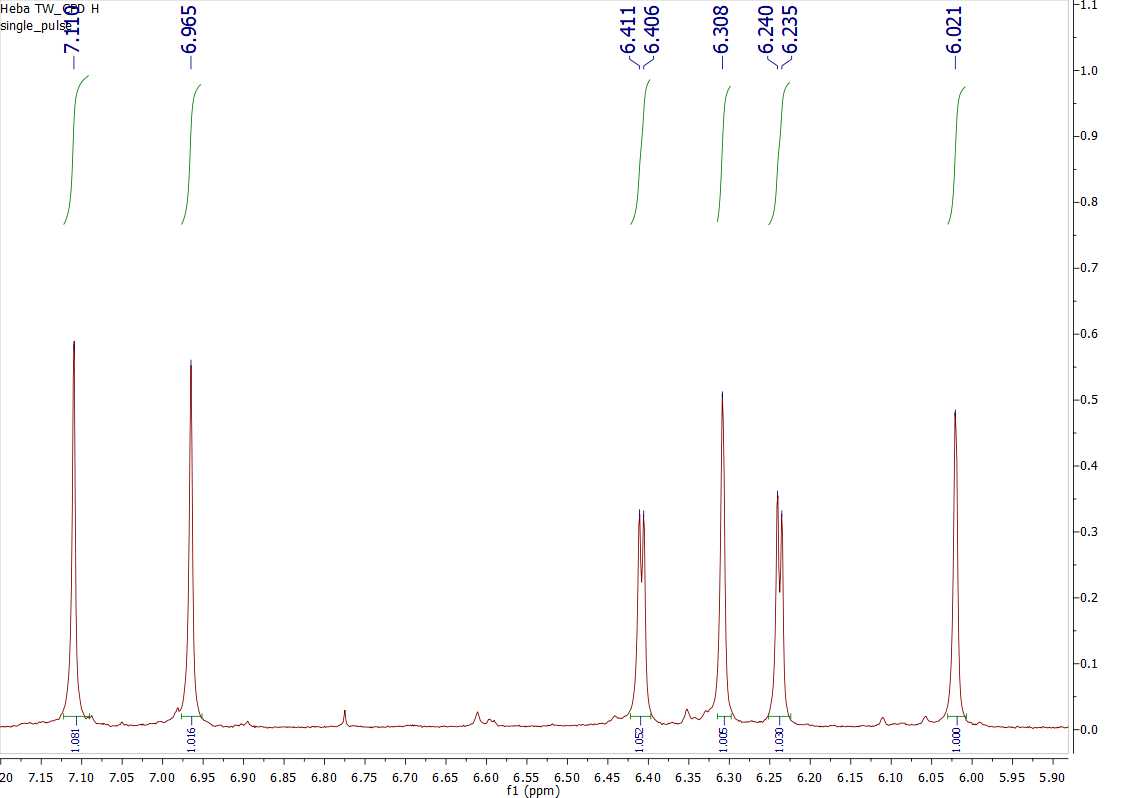


**Figure S18.** ^13^C NMR spectrum (100 MHz, CDCl_3_) of compound 5


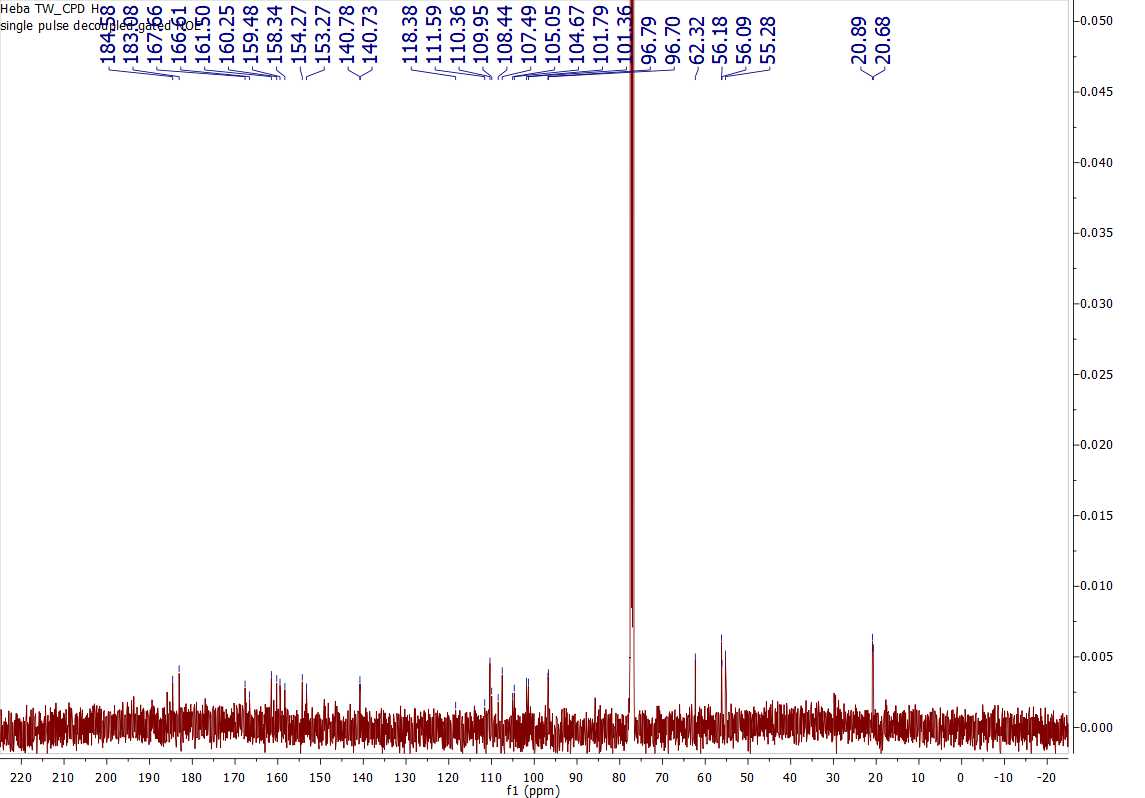

**Figure S19.** HMQC spectrum of compound 5


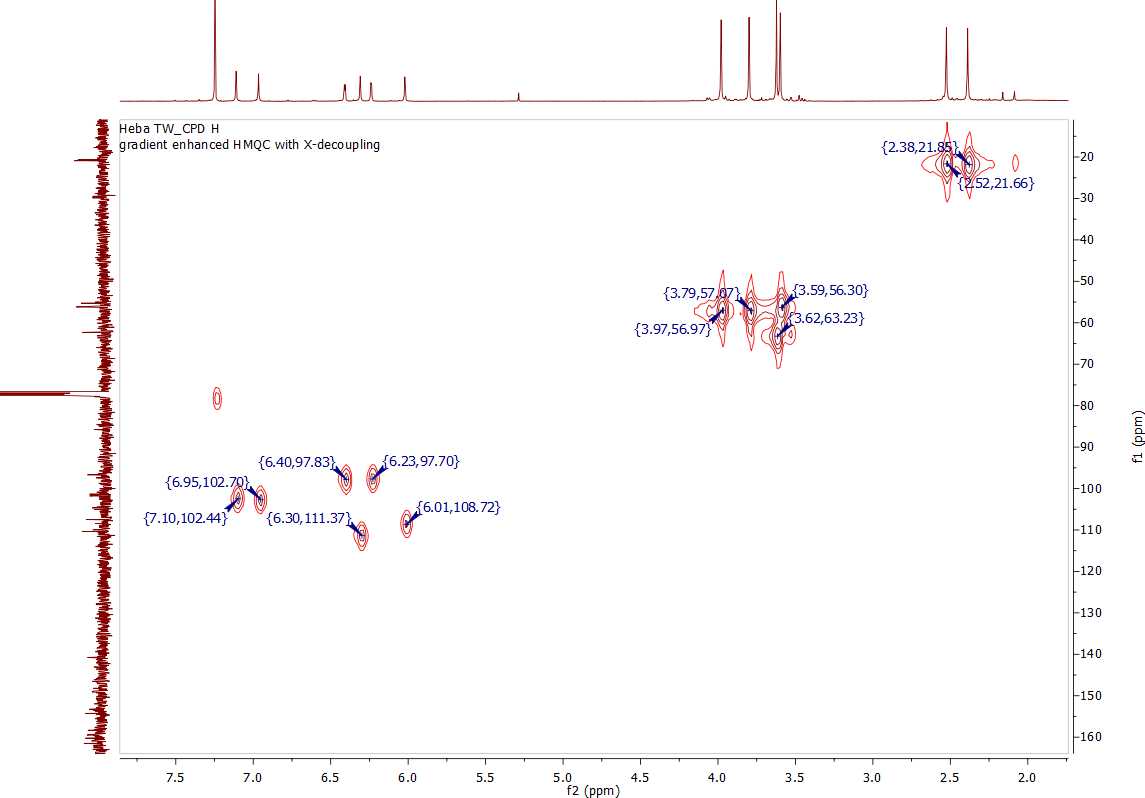


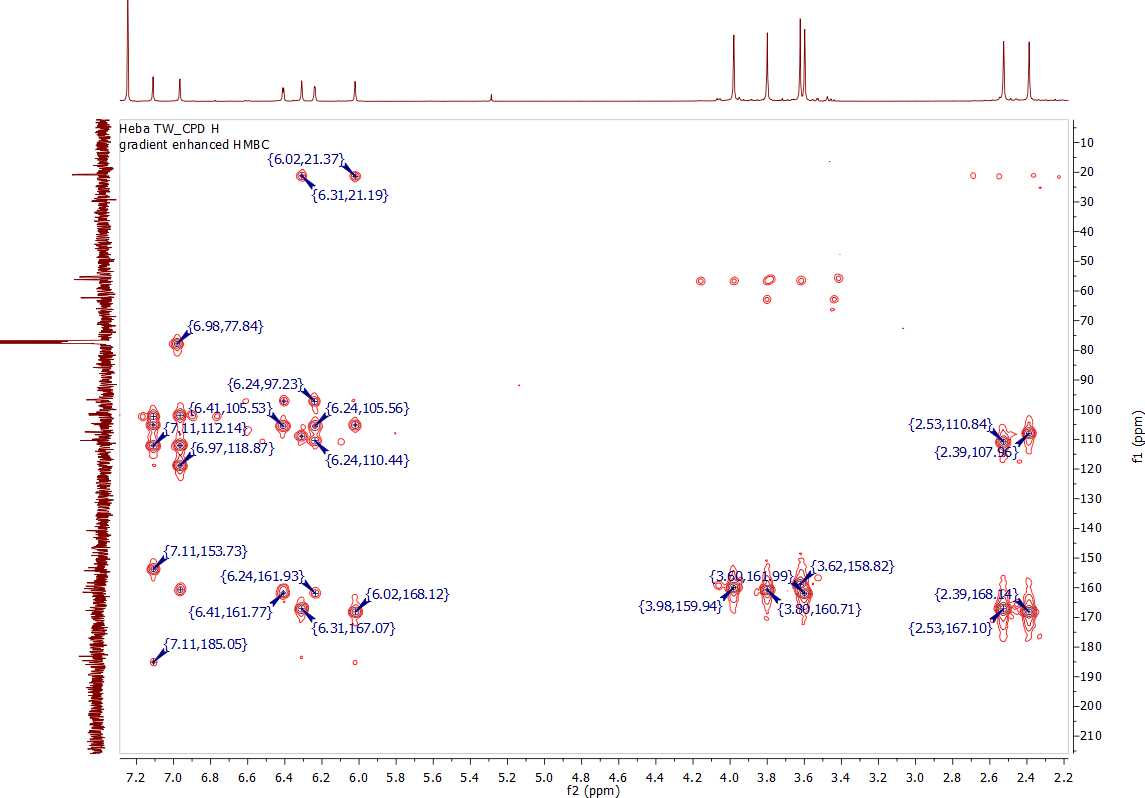
**Figure S20.** HMBC spectrum of compound 5

**Figure S**
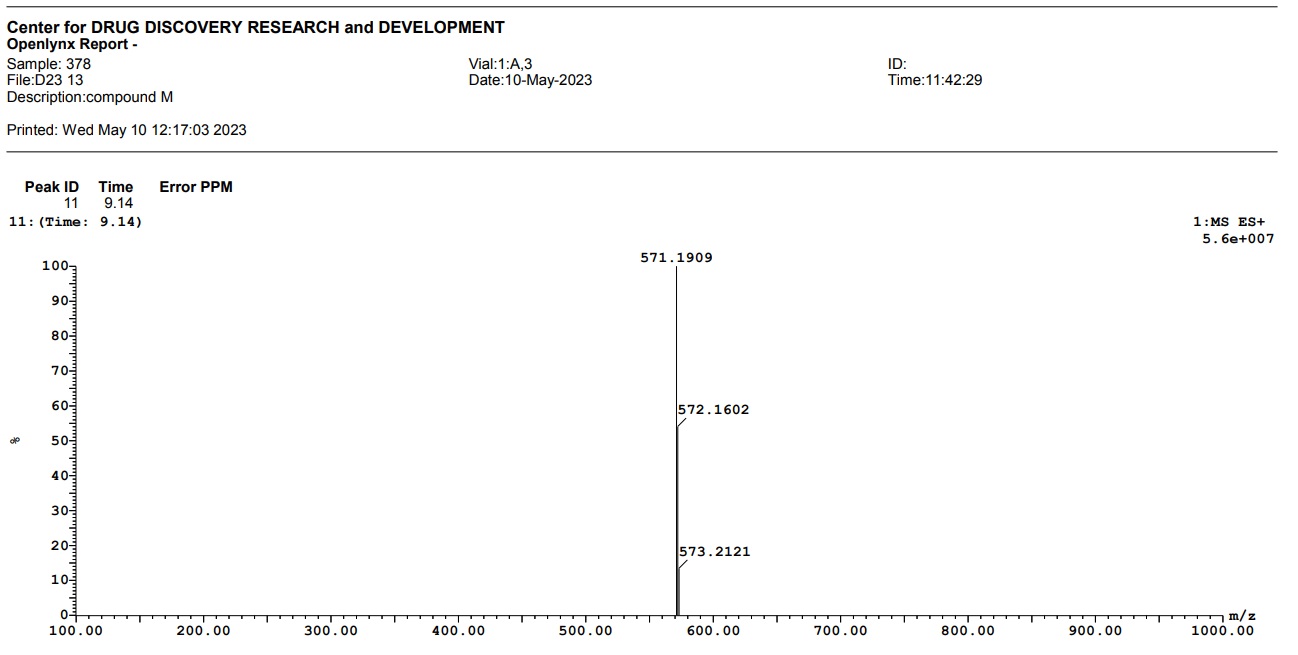
**21.** Positive ESI-MS spectrum of compound 6

**Figure S22.** ^1^H NMR spectrum (400 MHz, CDCl_3_) of compound 6
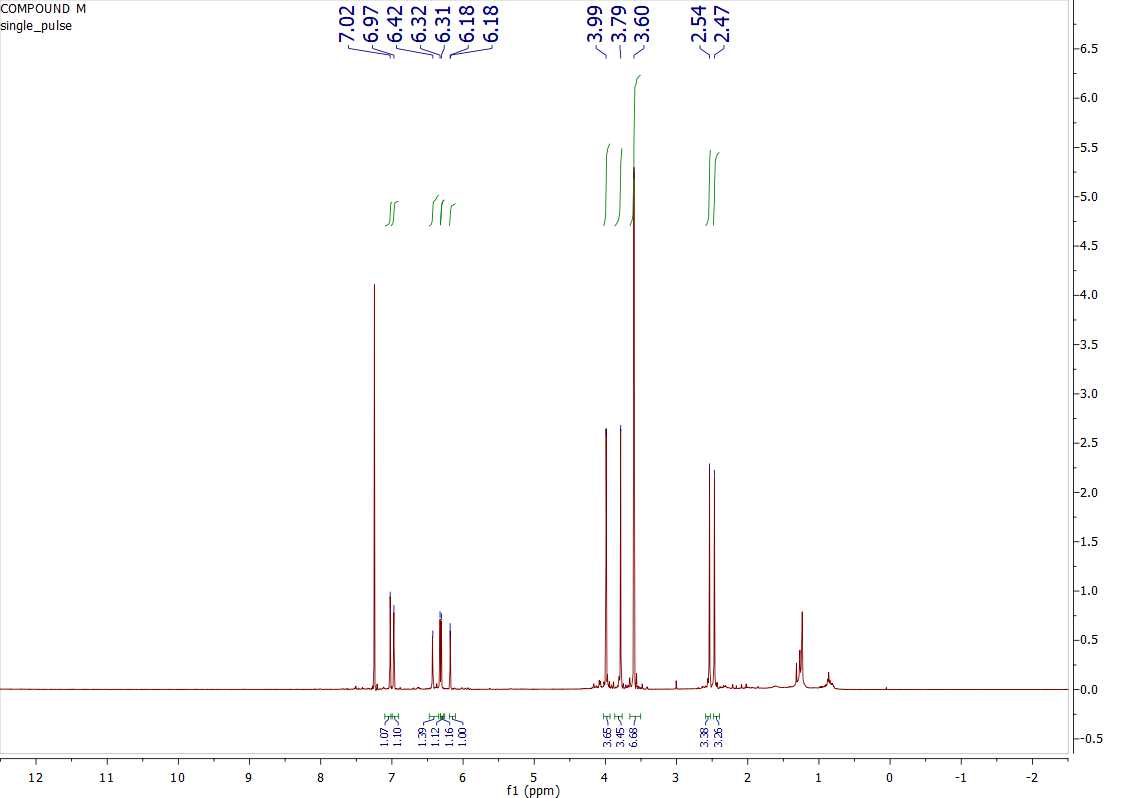

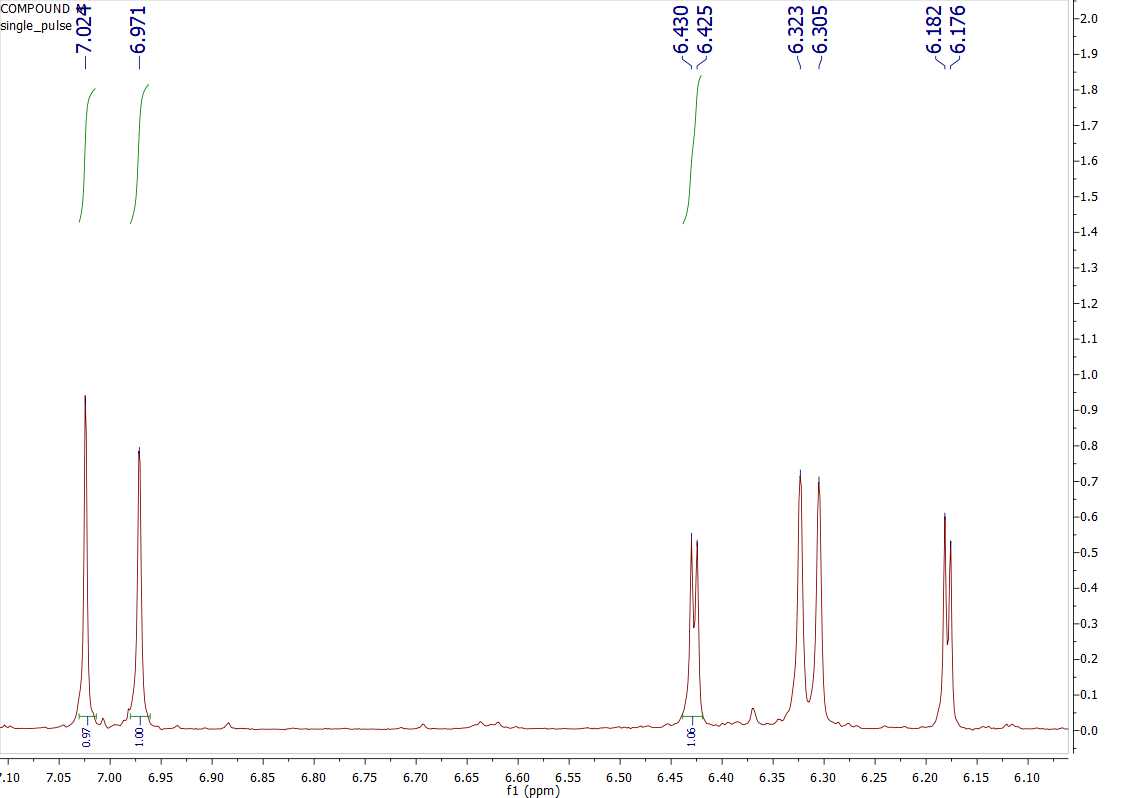


**Figure S23.** ^13^C NMR spectrum (100 MHz, CDCl_3_) of compound 6


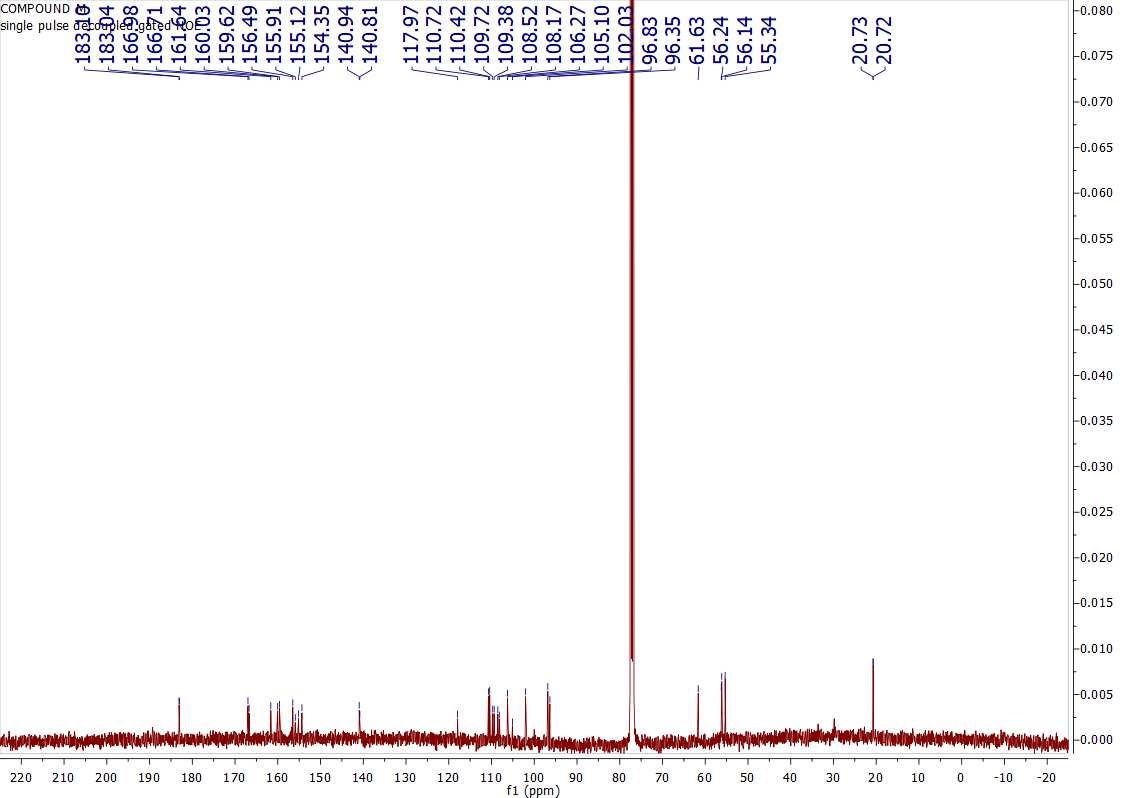

**Figure S24.** HMBC spectrum of compound 6

***.***


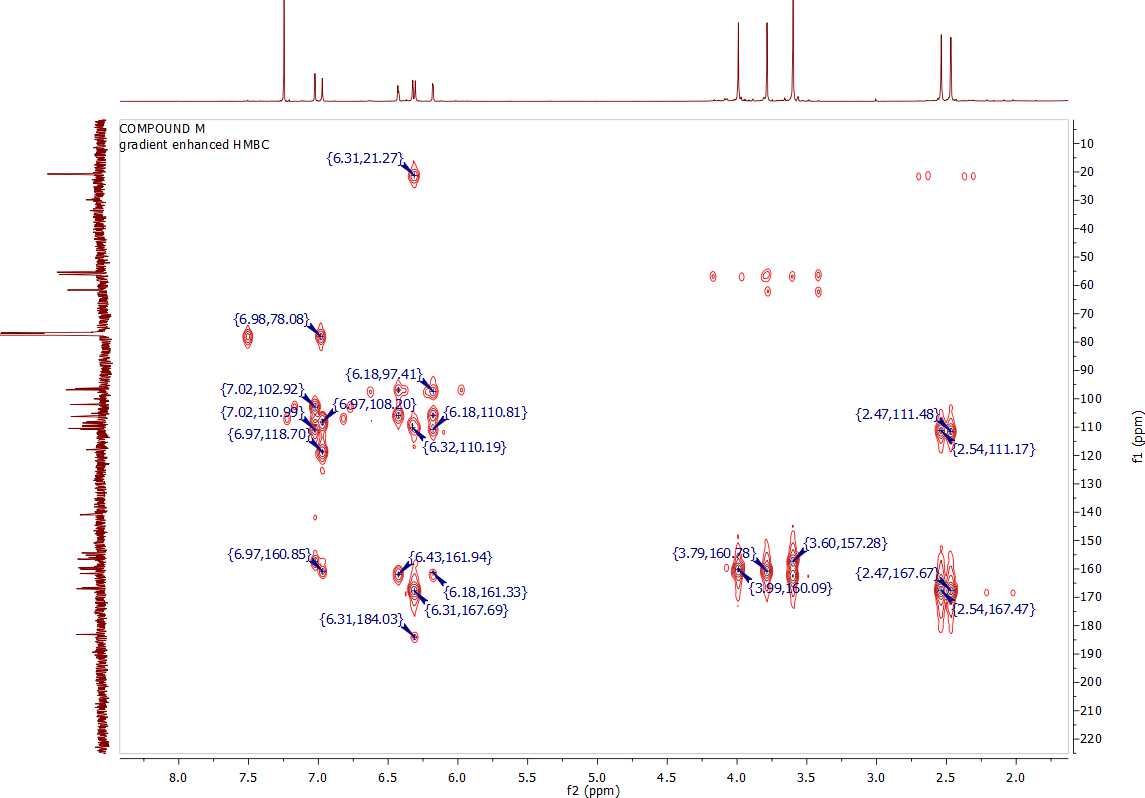

**Antimicrobial susceptibility test of tested samples**


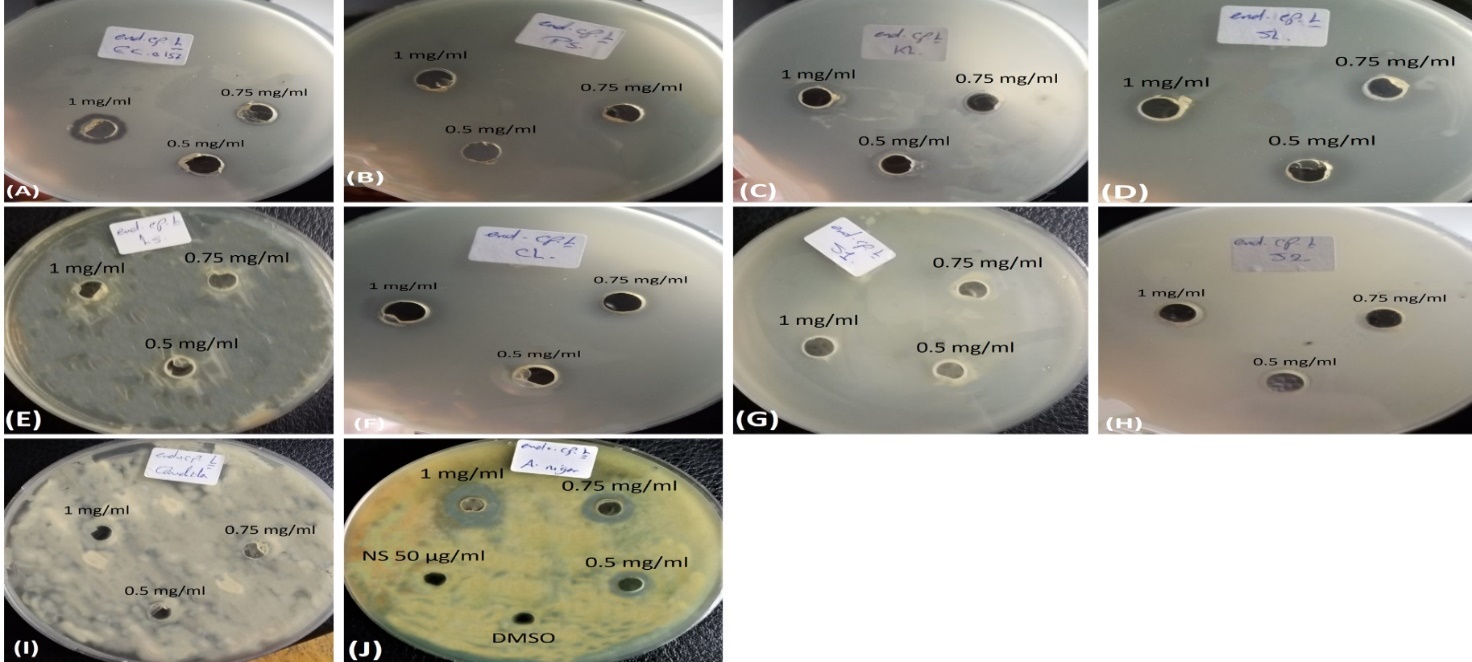


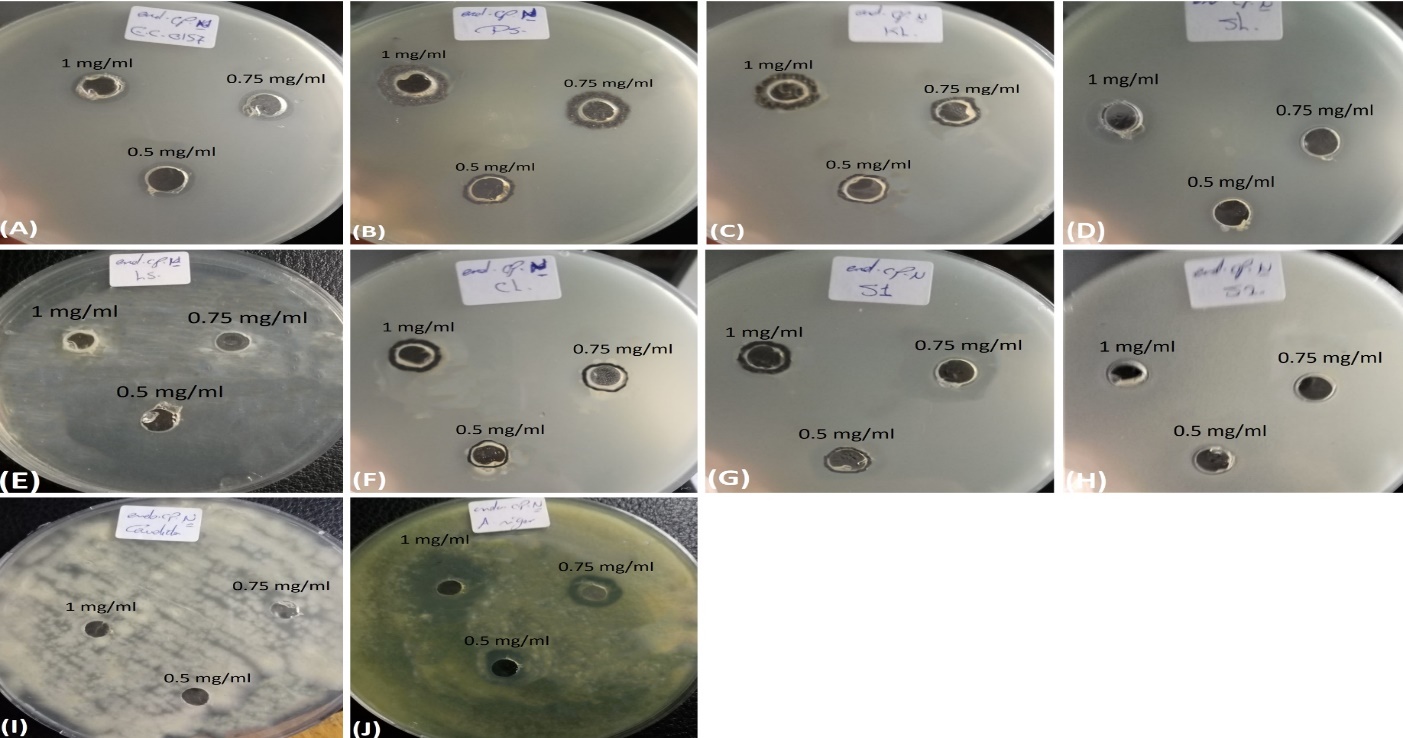
**Figure S25**. Inhibitory effect of different concentrations (1, 0.75 and 0.5 mg/ml) of ETOAC extract against reference strains**. (A) =** *Escherichia coli ATCC 8739 (E.C),* **(B)** = *Pseudomonas aeruginosa ATCC 9027 (*Ps.*),* **(C)** = *Klebsiella pneumonia ATCC 700603 (*K*),* **(D) =** *Salmonella enterica ATCC 14028* (SL.)*,* **(E)=** *Listeria monocytogenes ATCC 7644 (Ls.),* **(F) =** *Clostridium perfringens* ATCC 13124 (CL.), **(G)** *Staphylococcus aureus ATCC 25923* (S1)*,* **(H)** = *Streptococcus faecalis ATCC 8043* (S2)*,* **(I)***= Candida albicans ATCC 10231* (C)*, and* **(J)***= Aspergillus niger* ATCC 6275 (Asp.)*.*

**Figure S26**. Inhibitory effect of different concentrations (1, 0.75 and 0.5 mg/ml) of pure compound 1 against reference strains**. (A) =** *Escherichia coli ATCC 8739 (E.C),* **(B)** = *Pseudomonas aeruginosa ATCC 9027 (*Ps.*),* **(C)** = *Klebsiella pneumonia ATCC 700603 (*K*),* **(D) =** *Salmonella enterica ATCC 14028* (SL.)*,* **(E)=** *Listeria monocytogenes ATCC 7644 (Ls.),* **(F) =** *Clostridium perfringens* ATCC 13124 (CL.), **(G)** *Staphylococcus aureus ATCC 25923* (S1)*,* **(H)** = *Streptococcus faecalis ATCC 8043* (S2)*,* **(I)***= Candida albicans ATCC 10231* (C)*, and* **(J)***= Aspergillus niger* ATCC 6275 (Asp.)*.*


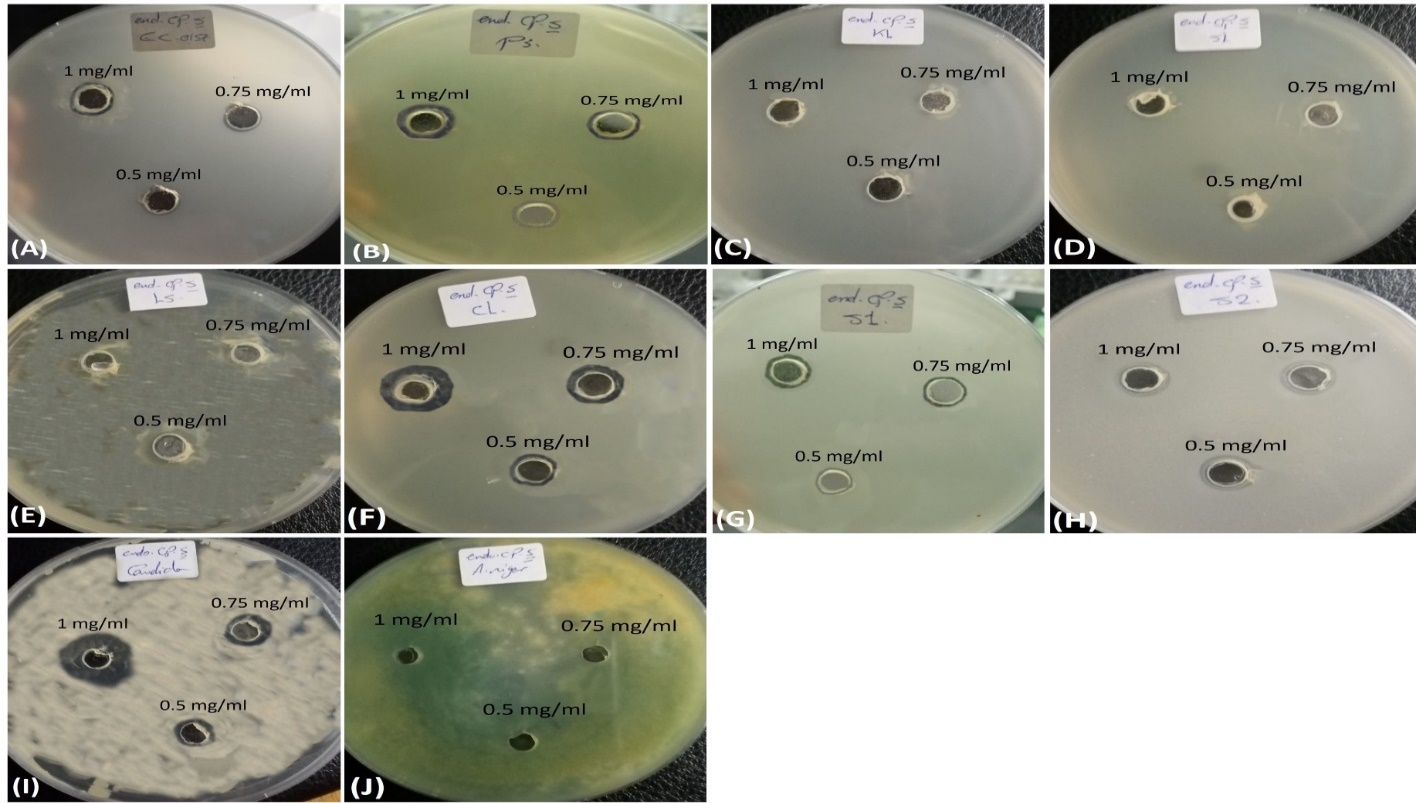


**Figure S27.** Inhibitory effect of different concentrations (1, 0.75 and 0.5 mg/ml) of pure compound 3 against reference strains. **(A) =** *Escherichia coli ATCC 8739 (E.C),* **(B)** = *Pseudomonas aeruginosa ATCC 9027 (*Ps.*),* **(C)** = *Klebsiella pneumonia ATCC 700603 (*K*),* **(D) =** *Salmonella enterica ATCC 14028* (SL.)*,* **(E)=** *Listeria monocytogenes ATCC 7644 (Ls.),* **(F) =** *Clostridium perfringens* ATCC 13124 (CL.), **(G)** *Staphylococcus aureus ATCC 25923* (S1)*,* **(H)** = *Streptococcus faecalis ATCC 8043* (S2)*,* **(I)***= Candida albicans ATCC 10231* (C)*, and* **(J)***= Aspergillus niger* ATCC 6275 (Asp.)*.*


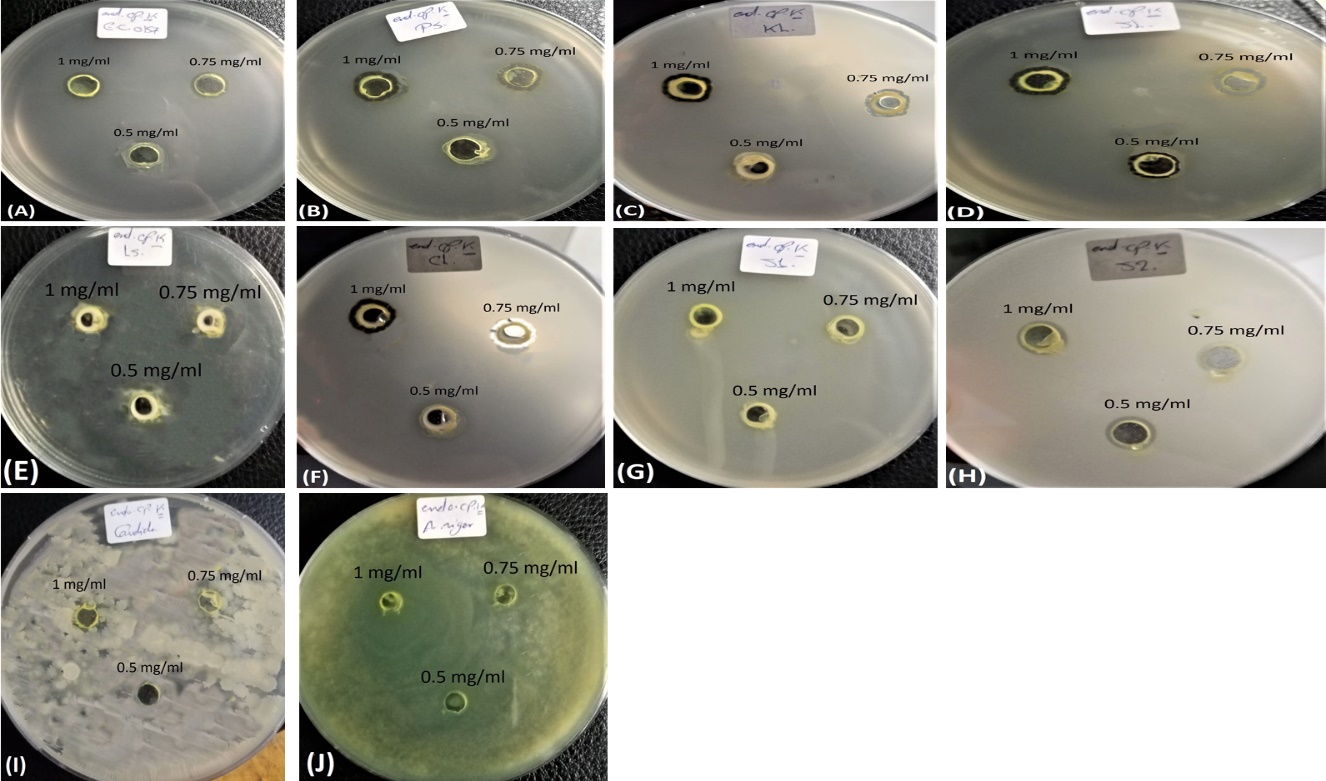


**Figure S28**. Inhibitory effect of different concentrations (1, 0.75 and 0.5 mg/ml) of pure compound 4 against reference strains**. (A) =** *Escherichia coli ATCC 8739 (E.C),* **(B)** = *Pseudomonas aeruginosa ATCC 9027 (*Ps.*),* **(C)** = *Klebsiella pneumonia ATCC 700603 (*K*),* **(D) =** *Salmonella enterica ATCC 14028* (SL.)*,* **(E)=** *Listeria monocytogenes ATCC 7644 (Ls.),* **(F) =** *Clostridium perfringens* ATCC 13124 (CL.), **(G)** *Staphylococcus aureus ATCC 25923* (S1)*,* **(H)** = *Streptococcus faecalis ATCC 8043* (S2)*,* **(I)***= Candida albicans ATCC 10231* (C)*, and* **(J)***= Aspergillus niger* ATCC 6275 (Asp.)*.*


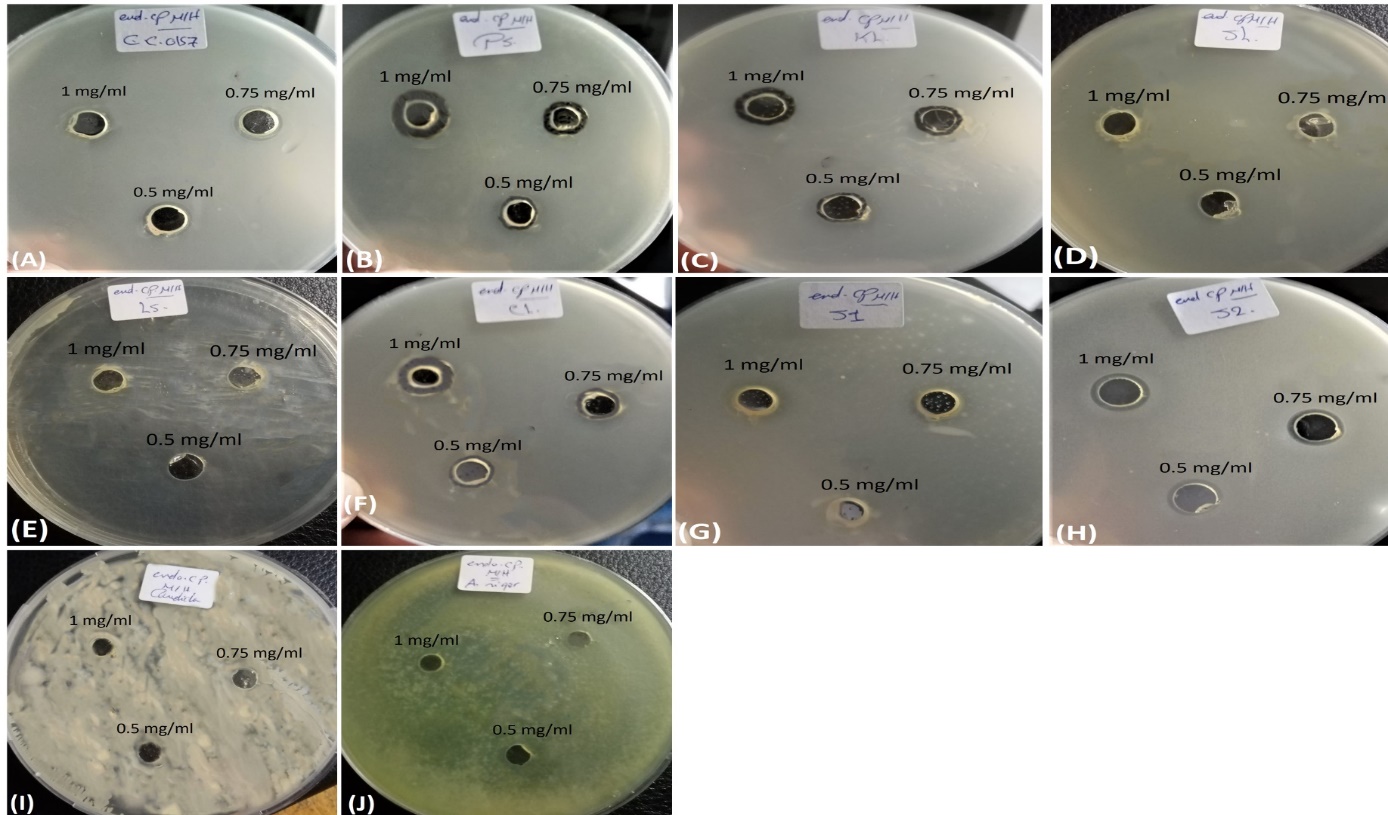


**Figure S29**. Inhibitory effect of different concentrations (1, 0.75 and 0.5 mg/ml) of pure compound 6 against reference strains**. (A) =** *Escherichia coli ATCC 8739 (E.C),* **(B)** = *Pseudomonas aeruginosa ATCC 9027 (*Ps.*),* **(C)** = *Klebsiella pneumonia ATCC 700603 (*K*),* **(D) =** *Salmonella enterica ATCC 14028* (SL.)*,* **(E)=** *Listeria monocytogenes ATCC 7644 (Ls.),* **(F) =** *Clostridium perfringens* ATCC 13124 (CL.), **(G)** *Staphylococcus aureus ATCC 25923* (S1)*,* **(H)** = *Streptococcus faecalis ATCC 8043* (S2)*,* **(I)***= Candida albicans ATCC 10231* (C)*, and* **(J)***= Aspergillus niger* ATCC 6275 (Asp.)*.*

**Antiproliferative activity**


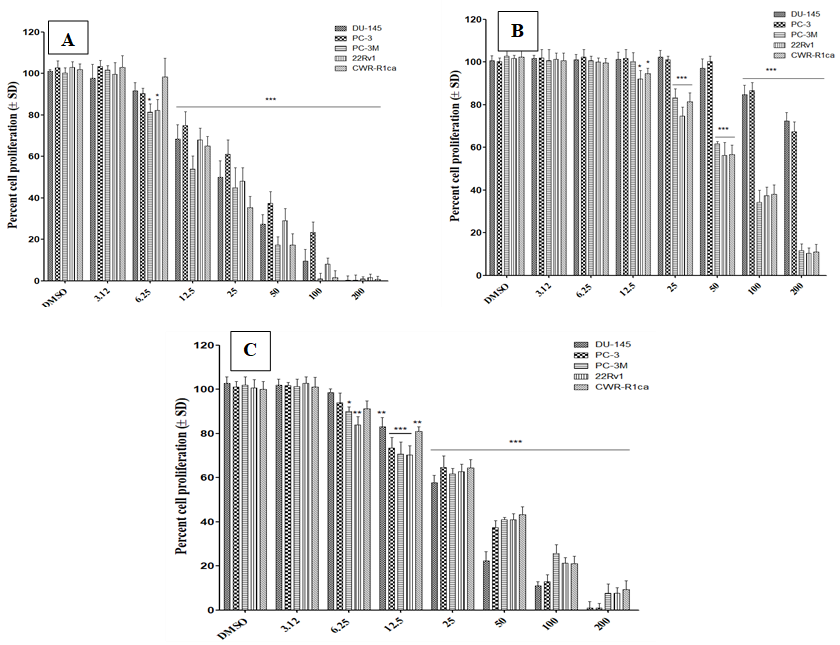


**Figure S30**. Concentration-response curve of isolated metabolites (1-3) on the proliferation of different prostate cancer cell lines. Bar graphs represent mean cell proliferation (±SD) and indicated concentrations. **A**) compound 1; **B**) compound 2; **C**) compound 3.


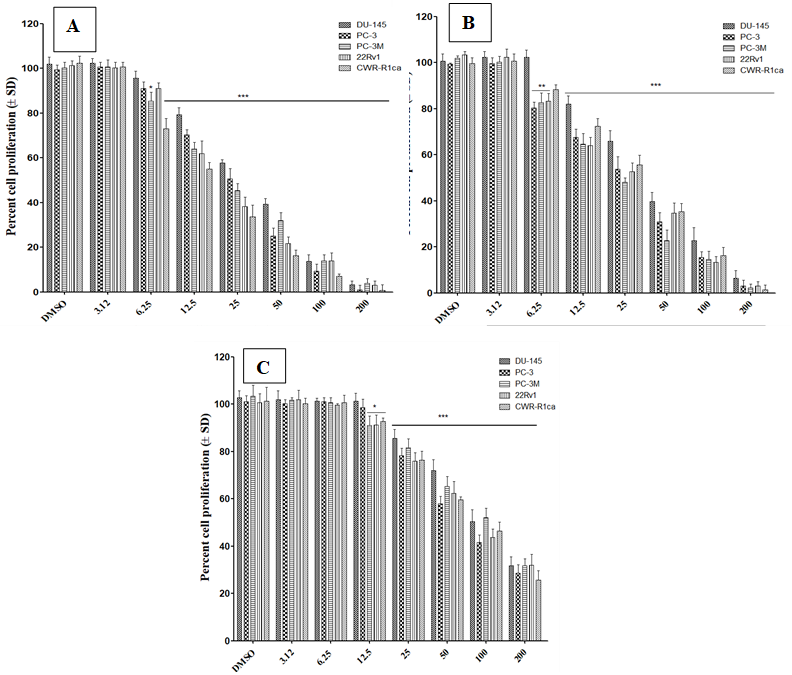


**Figure S31**. Concentration-response curve of isolated metabolites (4-6) on the proliferation of different prostate cancer cell lines. Bar graphs represent mean cell proliferation (±SD) and indicated concentrations. **A**) compound 4; **B**) compound 5; **C**) compound 6.
